# Changes in leukocytes and CRP in different treatments of major depression

**DOI:** 10.1007/s00702-026-03187-z

**Published:** 2026-07-02

**Authors:** Ammar Abo Hwach, Seda Nur Türker, Franziska Huster, Anastasia Gaspert, Alborz Bastami, Joana Wrasse, Stefan Bleich, Helge Frieling, Thomas Nickl-Jockschat, Hannah Benedictine Maier, Alexandra Neyazi

**Affiliations:** 1 Department of Psychiatry and Psychotherapy, Otto von Guericke University, Leipziger Straße 44, Magdeburg, 39120 Germany; 2 Department of Psychiatry, Social Psychiatry and Psychotherapy, Hannover Medical School, Hannover, Germany; 3 German Center for Mental Health (DZPG), partner site Halle-Jena-Magdeburg, Magdeburg, Germany; 4 Department of Psychiatry, Iowa Neuroscience Institute, University of Iowa, Iowa City, USA; 5 Center of Behavioral Brain Sciences, Otto von Guericke University, Magdeburg, Germany

**Keywords:** Neuroinflammation, Treatment-resistant depression (TRD), Neutrophil-lymphocyte ratio (NLR), Monocyte-lymphocyte ratio (MLR), Neutrophil-to-platelet ratio (NPR), Electroconvulsive therapy (ECT)

## Abstract

Neuroinflammatory mechanisms have long been implicated in the pathophysiology of depression, particularly in treatment-resistant depression (TRD). Peripheral immune markers such as monocyte-to-lymphocyte ratio (MLR), neutrophil-to-lymphocyte ratio (NLR), and neutrophil-to-platelet ratio (NPR) may reflect chronic or acute immune activation and could serve as potential biomarkers for treatment response. We analyzed differential blood cell counts and inflammatory ratios (MLR, NLR, NPR), as well as interleukin-6 (IL-6) and C-reactive protein (CRP) levels, in 196 patients with severe depression. Patients were grouped into unmedicated (UDC), TRD with medication only (TRD-M), and TRD with electroconvulsive therapy (TRD-ECT), and were assessed at baseline and after four weeks. TRD patients showed significantly higher monocyte and MLR levels than non-TRD patients. Within TRD, ECT responders exhibited lower monocyte and MLR values compared to non-responders. NLR levels were significantly higher in the ECT group. A positive correlation was found between NPR changes and symptom improvement based on Beck Depression Inventory-II scores (BDI-II). Our findings suggest that TRD is associated with a chronic inflammatory profile, and that ECT may induce a shift toward acute immune activation, potentially contributing to treatment response. MLR, NLR, and NPR may serve as potential biomarkers and should be evaluated in prospective studies with larger samples and detailed immune phenotyping.

**Supplementary Information:**

The online version contains supplementary material available at https://doi.org/10.1007/s00702-026-03187-z.

## Introduction

With a prevalence of 5% of people affected worldwide, unipolar depression is the leading cause of disability among all psychiatric disorders (Institute for Health Metrics and Evaluation (IHME) 2024). According to ICD-10 diagnostic criteria, depression is defined by core symptoms—depressed mood, loss of interest or anhedonia, and reduced energy or fatigue — accompanied by additional symptoms such as impaired concentration, reduced self-esteem, feelings of guilt, hopelessness, sleep disturbance, appetite changes, and suicidal ideation (World Health Organization 2019). Approximately 30% of patients develop treatment-resistant depression (TRD), which substantially contributes to the overall disease-related disability (McIntyre et al. 2023). TRD is also associated with a significantly higher risk of suicide (Gronemann et al. 2021), an increased demand for care, and substantial strain on medical resources (Zhdanava et al. 2020). In addition, TRD is linked to elevated risk of somatic comorbidities, including cardiovascular (Adekkanattu et al. 2023) and metabolic disorders (Godin et al. 2019). Even though some clinical risk factors for TRD are known, such as greater baseline symptom severity, comorbid anxiety disorders, longer episode duration and early age at onset, no reliable biomarkers have yet been identified. In this regard, immunological mechanisms are increasingly studied (Buoli et al. 2022; Mancuso et al. 2023; Lievanos-Ruiz and Fenton-Navarro 2025).

For a long time, the central nervous system (CNS) was considered immune-privileged due to unique mechanisms that tightly regulate, limit, or even suppress intracerebral immune activity, thereby preventing inflammation and protecting neural tissue from damage (Muldoon et al. 2013). However, growing evidence has challenged this view, demonstrating an association between neuroinflammation and depression. For example, peripheral inflammation — involving proinflammatory cytokines such as interleukin-1α and β (IL-1α and IL-1β), tumor necrosis factor-α (TNF-α), and interleukin-6 (IL-6) — has been identified as a biological risk factor for major depressive episodes through immune-to-brain communication pathways (Dantzer et al. 2008). It has therefore been hypothesized that neuroinflammatory processes may, at least in part, be reflected by peripheral inflammatory markers measurable in blood. In support of this, a meta-analysis confirmed depression as a pro-inflammatory state and demonstrated that elevations in peripheral markers such as CRP appear to reflect a right shift of the overall immune marker distribution rather than being driven solely by an inflamed subgroup (Osimo et al. 2020). Additionally, a statistically significant positive correlation between depression severity and C-reactive protein (CRP) levels was observed among female depressed subjects (Köhler-Forsberg et al. 2017).

As research into the relationship between neuroinflammation and depression has progressed, studies have focused on analyzing white blood cells, given their role in the body’s innate and adaptive immune response. One meta-analysis showed that individuals with depression tend to have higher levels of neutrophils and monocytes, along with an elevated neutrophil-to-lymphocyte ratio (NLR), suggesting a shift toward innate immune activation (Sørensen et al. 2023). NLR was also found to be elevated in depressed patients, underlining its potential as a peripheral marker of inflammation (Zhou et al. 2025). Together, these results underscore the potential importance of such markers in understanding the biological basis of depression.

Antidepressant medication (Wang et al. 2019), and electroconvulsive therapy (ECT) have been suggested to have anti-inflammatory properties in patients with depression (Lehtimäki et al. 2008). ECT is regarded as the most effective treatment method for severe depression, especially for TRD (UK ECT Review Group 2003). ECT has been shown to induce transient changes in peripheral white blood cell (WBC) counts, indicating a possible interaction with the immune system (Chaturvedi et al. 2001). This acute, short-lived leukocytosis following a therapy session is primarily driven by increases in neutrophil counts and is typically considered a non-specific physiological stress response mediated by the hypothalamic-pituitary-adrenal (HPA) axis activation and muscle contractions, rather than a direct immune modulation related to the antidepressant effect (Fluitman et al. 2011).

The neutrophil-to-platelet ratio (NPR) has recently emerged as a novel inflammatory marker that effectively accounts for the intensity of the acute inflammatory response, as reflected by neutrophils, while also considering preexisting chronic inflammatory states, which are represented by platelets (Wei et al. 2017). Although NPR has shown promise in other contexts (Yamamoto-Furusho and Mendieta-Escalante 2020; Zhu et al. 2025; Nguyen et al. 2026), only one study (Geng et al. 2024) has so far explored its role as an inflammation marker in depression by analyzing adolescents with depression undergoing ECT. It remains unclear whether significant changes in NPR occur between patients who respond to antidepressant treatment and those who do not. Considering growing evidence on the link between inflammation and depression, and to address the gap in literature, we sought to investigate whether NPR, NLR, and Monocyte-lymphocyte ratio (MLR), differential blood count, and CRP levels may also serve as a useful marker in depression, particularly in the context of treatment response.

## Methods

### Study design and population

This study included patients treated for severe depressive disorder at the Department of Psychiatry, Social Psychiatry and Psychotherapy at Hannover Medical School (MHH). Data were obtained from the Northern German Electroconvulsive Therapy Outcome Registry (NEKTOR – Norddeutsches Elektrokonvulsionstherapie Outcome Register) at MHH. The NEKTOR registry and related outcomes have been reported in previous publications (Moschny et al. 2020b; Maier et al. 2022, 2023; Schülke et al. 2023; Gaspert et al. 2025; Houjaije et al. 2025).

Patients were aged 18 to 75 years and were admitted as out- or inpatients at the time of inclusion in NEKTOR. Each patient had a primary diagnosis of either a major depressive episode or recurrent severe depressive disorder, classified according to the 10th revision of the International Statistical Classification of Diseases and Related Health Problems (ICD-10) (World Health Organization 2019). All diagnoses were confirmed using the Structured Clinical Interview for DSM (SCID), conducted by trained psychiatrists. Depression was considered as TRD when two antidepressants, administered in adequate dosages, fail to achieve a significant improvement in symptoms (McIntyre et al. 2023). Exclusion criteria included a secondary diagnosis of schizophrenia, active substance use disorders, treatment with immunomodulatory drugs or corticosteroids, an acute infection with leukocytosis and/or elevated CRP, or a missing differential blood count. Documented F10–F19 diagnoses in the study sample reflect lifetime or anamnestic diagnoses rather than acute substance use at the time of inclusion.

In total, 246 patients were included at baseline. After applying exclusion criteria, 196 patients remained in the study. The reduction from 246 to 196 patients was due to the application of predefined exclusion criteria rather than clinical dropout; the majority of exclusions resulted from missing differential blood counts at baseline. Patients were categorized into two main groups: (1) “Unmedicated depressive Controls” (UDC), consisting of patients with a severe depressive episode who were free of antidepressant medication at baseline for at least five half-lives prior to inclusion (*n* = 36). These patients were either antidepressant-naïve at the time of admission or had discontinued prior medication before inclusion. Following baseline assessment, all UDC patients received antidepressant treatment according to clinical need and guideline-based care. (2) The “Treatment-Resistant Depression” (TRD) group, including all patients diagnosed with treatment-resistant depression. The TRD group was further divided into (2a) “TRD with medication” (TRD-M), comprising severely depressed patients receiving antidepressant medication without ECT, although ECT was clinically indicated in some cases but not administered (e.g., due to patient preference or other clinical considerations) (*n* = 82), and (2b) “TRD with Electroconvulsive Therapy” (TRD-ECT), consisting of severely depressed patients treated with both antidepressant medication and ECT (*n* = 78).

All patients were assessed at two specific time points: upon hospital admission and after four weeks. The four-week interval was based on the TRD-ECT group, as the final ECT session occurs around the four-week mark. Therefore, the second observation point was set at four weeks for all patients. At each observation point, assessments included psychometric testing and venous blood sampling. Depressive symptoms were evaluated through both self- and clinician-rated assessments using the Beck Depression Inventory 2nd Edition (BDI-II) (Beck et al. 2011) and the Montgomery–Åsberg Depression Rating Scale (MADRS) (Montgomery and Asberg 2012). ‘Remission’ was defined as a total MADRS score below 10, and ‘Response’ was defined as a ≥ 50% reduction in the baseline MADRS score at week 4.

All patients provided written informed consent prior to study inclusion. This study was approved by the Ethics Committee of Hannover Medical School (2842 − 2015) and adhered to all ethical guidelines of the Declaration of Helsinki (1964) as well as its subsequent amendments.

### Blood sampling and analysis

After collection, samples were sent to the Central Laboratory of Hannover Medical School (Zentrallabor der MHH) and analyzed on the same day. Differential blood counts, CRP, and IL-6 were processed without delay, with no differences in processing time between groups. Storage at 4 °C applied only to samples reserved for subsequent specialized analyses not reported in this study. IL-6 was measured at baseline only, whereas CRP and the differential blood count were assessed at both time points.

CRP and IL-6 levels were measured using the cobas 8000 system (Roche Diagnostics). IL-6 was quantified with an electrochemiluminescence immunoassay (ECLIA) and CRP levels were determined using an immunoturbidimetric assay. Differential blood counts were analyzed with the XN9000 system (Sysmex) using flow cytometry.

### Electroconvulsive therapy (ECT)

NEKTOR patients assigned to TRD-ECT received ECT sessions three times weekly, with a total of 10 ± 4 treatments. ECT was administered using the brief-pulse Thymatron^®^ System IV (Somatics, Lake Bluff, Illinois, USA), and stimulus intensity was determined using the age-based method. Treatment was initiated with right unilateral electrode placement and switched to bitemporal placement after non-response at the sixth session. Anesthesia consisted of methohexital or propofol with remifentanil, and muscle relaxation was achieved with succinylcholine or mivacurium. Seizures were monitored by two-channel frontomastoidal electroencephalography (EEG), while motor seizure activity was assessed using a blood pressure cuff inflated on the right lower leg prior to muscle relaxation. Seizure quality was evaluated on the basis of electroencephalographic seizure duration and motor seizure activity. Treatment parameters were adjusted according to seizure quality and clinical response.

### Statistical analysis

For statistical analysis, we used RStudio version 2023.12.1 + 402. Data are presented as mean ± SD for continuous variables and as n (%) for categorical variables. Normality was assessed using the Shapiro–Wilk test. For normally distributed data, two-group comparisons were performed using t-tests, whereas comparisons across more than two groups were performed using analysis of variance (ANOVA). Non-normally distributed data were analyzed using the Mann–Whitney U test (two groups) or the Kruskal–Wallis H test (more than two groups), followed by Dunn’s test for pairwise comparisons with Bonferroni correction for multiple testing. Categorical variables were analyzed using the chi-square test when expected cell counts were ≥ 5; otherwise, Fisher’s exact test was used. Paired data were analyzed using the Wilcoxon signed-rank test. Correlations between cell count percentages and demographic, clinical, and blood parameters were assessed using Spearman’s rank correlation, applying False Discovery Rate (FDR) correction (Benjamini and Hochberg 1995). All tests were two-sided, and p-values < 0.05 were considered statistically significant.

Linear mixed models were fitted using the lme4 package in RStudio. Participant ID was included as a random intercept to account for repeated measures. Age, sex, body mass index (BMI), and smoking status were included as random effects based on previous literature (Helman and Rubenstein 1975; Carel and Eviatar 1985; Schwartz and Weiss 1991). For models analyzing neutrophils (%), lithium use was included as an additional covariate given its known effect on neutrophil counts (Rothstein et al. 1978; Boggs and Joyce 1983; Joyce 1984). To further confirm that lithium did not confound our results, we repeated the analyses of NLR, NPR and neutrophils (%) after excluding all lithium users. All models were fitted using restricted maximum likelihood (REML = TRUE).

For each outcome, we tested the three-way interaction between timepoint (baseline vs. four weeks), MADRS response (responder vs. non-responder), and (i) participant group (TRD vs. UDC), as well as (ii) ECT treatment (yes vs. no). Satterthwaite’s method (via lmerTest) was used to assess the overall significance of the interaction terms in a Type III ANOVA. When the overall interaction was significant, post hoc multiple comparisons were performed using the emmeans package with Tukey correction.

In these models, TRD encompassed all patients clinically diagnosed with treatment-resistant depression, irrespective of treatment type (TRD-M and TRD-ECT). For analyses addressing the impact of ECT, patients receiving ECT (TRD-ECT) were compared with patients not receiving ECT. Due to the small number of responders in the UDC group (*n* = 2), UDC was not considered in the interpretation of responder-related interaction effects. Cell count percentages from the differential blood count were used in correlation analyses and linear mixed models to reduce the influence of inter-individual variability in total leukocyte counts and improve comparability across participants. Supplementary analyses using absolute counts are reported in the Online Resource (Table S12-S13).

## Results

### Patient characteristics

Demographic and clinical characteristics are summarized in Table 1.

**Table 1:** Demographic and clinical parameters

					post-hoc		
Variables	TRD-M (n = 82)	TRD-ECT (n = 78)	UDC (n = 36)	p-value	TRD-M/TRD-ECT	TRD-M/UDC	UDC/TRD-ECT
Age, years	44.2 (± 15.5)	51.9 (± 13.9)	35.4 (± 14.9)	**< 0.001**	**0.008**	**0.014**	**< 0.001**
Duration of current depressive episode, weeks	135.4 (± 186.3)	115.5 (± 167.4)	97.1 (± 106.2)	0.906			
Female, n(%)	36 (43.9)	48 (61.5)	15 (41.7)	**0.042**	0.114	1.000	0.225
Body Mass Index, kg/m²	27.6 (± 5.7)	26.4 (± 5.4)	25.9 (± 6.1)	0.148			
Smoker, n (%)	37 (45.1)	48 (61.5)	18 (50)	0.109			
Current suicidality, n (%)	27 (37.0)	27 (36.5)	11 (32.4)	0.889			
MADRS (baseline)	30.1 (± 7.6)	31.6 (± 9.4)	27.6 (± 8.1)	0.075			
MADRS (week 4)	25.9 (± 8.7)	15.5 (± 11.2)	22.9 (± 8.7)	**< 0.001**	**< 0.001**	0.369	**0.007**
MADRS responders, n/N (%)	6/46 (13.0)	27/51 (52.9)	2/27 (7.4)	**< 0.001**	**< 0.001**	1.000	**< 0.001**
BDI-II (baseline)	36.1 (± 10.7)	38.5 (± 10.2)	31 (± 10.6)	**0.003**	0.332	**0.048**	**0.002**
BDI-II (week 4)	32.3 (± 10.4)	21.4 (± 15.3)	26.6 (± 11.2)	**0.001**	**< 0.001**	0.126	0.248
Cortisol, µg/dL (baseline)	14.7 (± 5.4)	15.9 (± 4.8)	14.1 (± 4.7)	0.176			
Cortisol, µg/dL (week 4)	14.6 (± 5.0)	14.7 (± 6.3)	14.44 (± 4.7)	0.988			

Patients’ characteristics presented as mean ± standard deviation (SD) or absolute quantity and percentage (n [%]); For responder analyses, N denotes the number of participants with available data for the respective scale percentages are calculated using this N

*MADRS* montgomery-Åsberg depression rating scale, *BDI-II * beck depression inventory-II, *UDC* unmedicated depressive controls, *TRD-M* treatment-resistant depression (medication only), *TRD-ECT * treatment-resistant depression (electroconvulsive therapy)

Significant p-values are highlighted in bold font

Regarding age distribution, patients in the UDC group were significantly younger than those in the TRD-M (*p* = 0.014) and the TRD-ECT group (*p* < 0.001). Additionally, patients in the TRD-ECT group were older than those in the TRD-M group (*p* = 0.008). A slight overall difference in sex distribution was observed between groups (*p* = 0.042), with a higher proportion of women in the TRD-ECT group compared to the other groups. No significant differences were detected for body mass index (BMI) or smoking status.

Medication use within the TRD group is shown in Table 2. Detailed mixed-model outputs (Type III ANOVA) and Tukey-adjusted post hoc pairwise comparisons are provided in the Online Resource 1 (Tables S1-S13).

**Table 2:** Medication treatment in the TRD group

Variables	TRD-M	TRD-ECT	*p*-value
SSRI/SNRI, n (%)	48 (58.5)	43 (55.1)	0.783
Typical antipsychotics, n (%)	7 (8.5)	19 (24.4)	**0.013**
Atypical antipsychotics, n (%)	23 (28.0)	46 (59.0)	**< 0.001**
Tricyclic & tetracyclic antidepressants, n (%)	7 (8.5)	16 (20.5)	**0.053**
Other antidepressants, n (%)	38 (46.3)	20 (25.6)	**0.011**
Lithium, n (%)	6 (7.3)	15 (19.2)	**0.046**
Benzodiazepines and Z-drugs, n (%)	11 (13.4)	21 (26.9)	0.053

Other antidepressants include NaSSA, Tianeptine, Bupropion, Agomelatine and Trazodone

*TRD-M* treatment-resistant depression (medication only), *TRD-ECT* treatment-resistant depression (electroconvulsive therapy)

Significant p-values are highlighted in bold font

### Depression severity and response

At baseline, MADRS scores did not differ significantly between groups (*p* = 0.075). After four weeks, significant group differences emerged (*p* < 0.001). Post hoc comparisons showed lower MADRS scores in the TRD-ECT group compared to TRD-M (*p* < 0.001) and UDC (*p* = 0.007), while no difference was seen between TRD-M and UDC (*p* = 0.369). Within-group comparisons showed reductions in MADRS scores in all groups after four weeks (TRD-M: Δ = −3.4 ± 9.1, *p* = 0.014; TRD-ECT: Δ = −16.7 ± 14.5, *p* < 0.001; UDC: Δ = −3.8 ± 7.2, *p* = 0.011). Further details are provided in Table 3.

**Table 3:** Within-group changes in psychometric measures (MADRS, BDI-II) from baseline to week 4

Group	Baseline	Week 4	Δ	p-value	Effect size (Cohen’s d) [95% CI]
*MADRS*					
TRD-M	29.4 (± 6.7)	25.9 (± 8.7)	−3.4 (± 9.1)	**0.014**	−0.38 [−0.67; −0.07]
TRD-ECT	32.3 (± 9.4)	15.5 (± 11.2)	−16.7 (± 14.5)	**< 0.001**	−1.15 [−1.50; −0.79]
UDC	26.7 (± 8.3)	22.9 (± 8.7)	−3.8 (± 7.2)	**0.011**	−0.53 [−0.92; −0.12]
*BDI-II*					
TRD-M	34.0 (± 9.3)	31.8 (± 10.0)	−2.3 (± 7.0)	**0.035**	−0.33 [−0.62; −0.02]
TRD-ECT	39.7 (± 10.1)	21.4 (± 15.3)	−18.3 (± 13.9)	**< 0.001**	−1.32 [−1.68; −0.94]
UDC	31.7 (± 11.3)	26.5 (± 11.4)	−5.2 (± 9.6)	**0.009**	−0.55 [−0.95; −0.14]

Data are presented as mean ± standard deviation (SD). *Δ = *Week 4 – Baseline

*MADRS* Montgomery-Åsberg depression rating scale, *BDI-II* beck depression inventory-II, *UDC* unmedicated depressive controls, *TRD-M* treatment-resistant depression (medication only), *TRD-ECT* treatment-resistant depression (electroconvulsive therapy)

Significant p-values are highlighted in bold font

Baseline BDI-II scores differed between groups (*p* = 0.003): UDC had lower baseline BDI-II scores than TRD-M (*p* = 0.048) and TRD-ECT (*p* = 0.002), while TRD-M and TRD-ECT did not differ (*p* = 0.332). After four weeks, BDI-II scores in TRD-ECT were lower than those in TRD-M (*p* < 0.001). Within-group analyses showed reductions in BDI-II over four weeks (TRD-M: Δ = −2.3 ± 7.0, *p* = 0.035; TRD-ECT: Δ = −18.3 ± 13.9, *p* < 0.001; UDC: Δ = −5.2 ± 9.6, *p* = 0.009). Further details are provided in Table 3.

### White blood cell count percentage

**Fig. 1:**
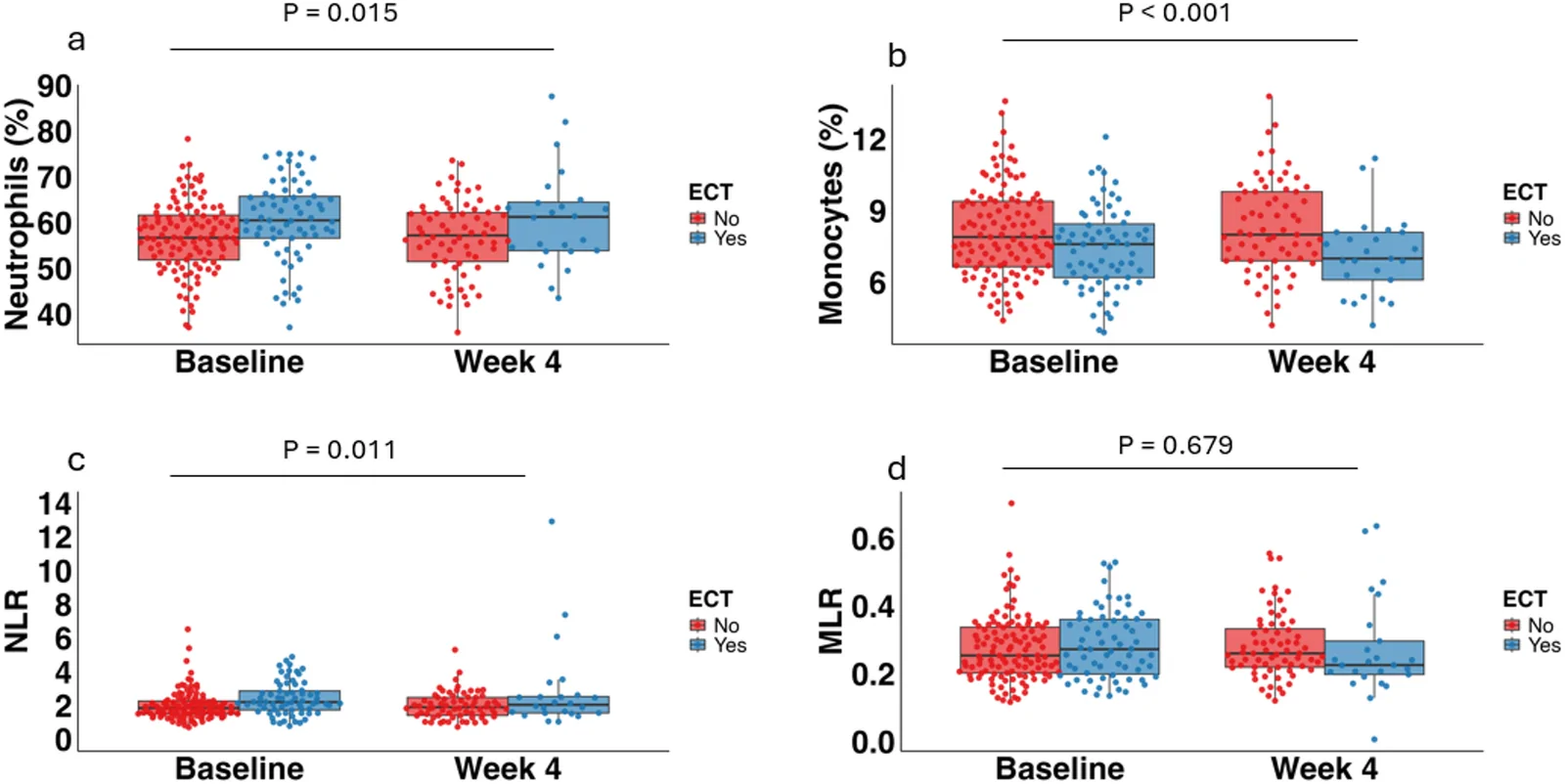
Peripheral inflammatory markers by ECT exposure over time. Boxplots with individual data points show values at baseline and week 4 stratified by ECT exposure (No vs. Yes). Panels: **a** Neutrophils (%), **b** Monocytes (%), **c** Neutrophil-to-lymphocyte ratio (NLR), and **d** Monocyte-to-lymphocyte ratio (MLR). P values indicate the main effect of ECT in the linear mixed models (averaged across time points): **a** *p* = 0.015, **b** *p* < 0.001, **c** *p* = 0.011, and **d** *p* = 0.679

At baseline, neutrophils (%), lymphocytes (%), eosinophils (%), and basophils (%) levels did not differ between TRD and UDC (model-derived pairwise comparisons by timepoint; neutrophils: *p* = 0.783; lymphocytes: *p* = 0.778; eosinophils: *p* = 0.920; basophils: *p* = 0.879). Neutrophils (%) levels differed by ECT exposure, with higher values in patients receiving ECT compared with non-ECT patients (main effect of ECT: *p* = 0.015; Fig. 1a). When the analysis was repeated excluding patients receiving lithium, the main effect of ECT on neutrophils (%) remained significant (main effect of ECT: *p* = 0.039). In separate cross-sectional models the difference between ECT and non-ECT patients was not statistically significant at baseline (*p* = 0.063) but reached significance at week 4 (*p* = 0.019), suggesting that the overall effect was primarily driven by the post-treatment time point rather than pre-existing group differences.

**Fig. 2:**
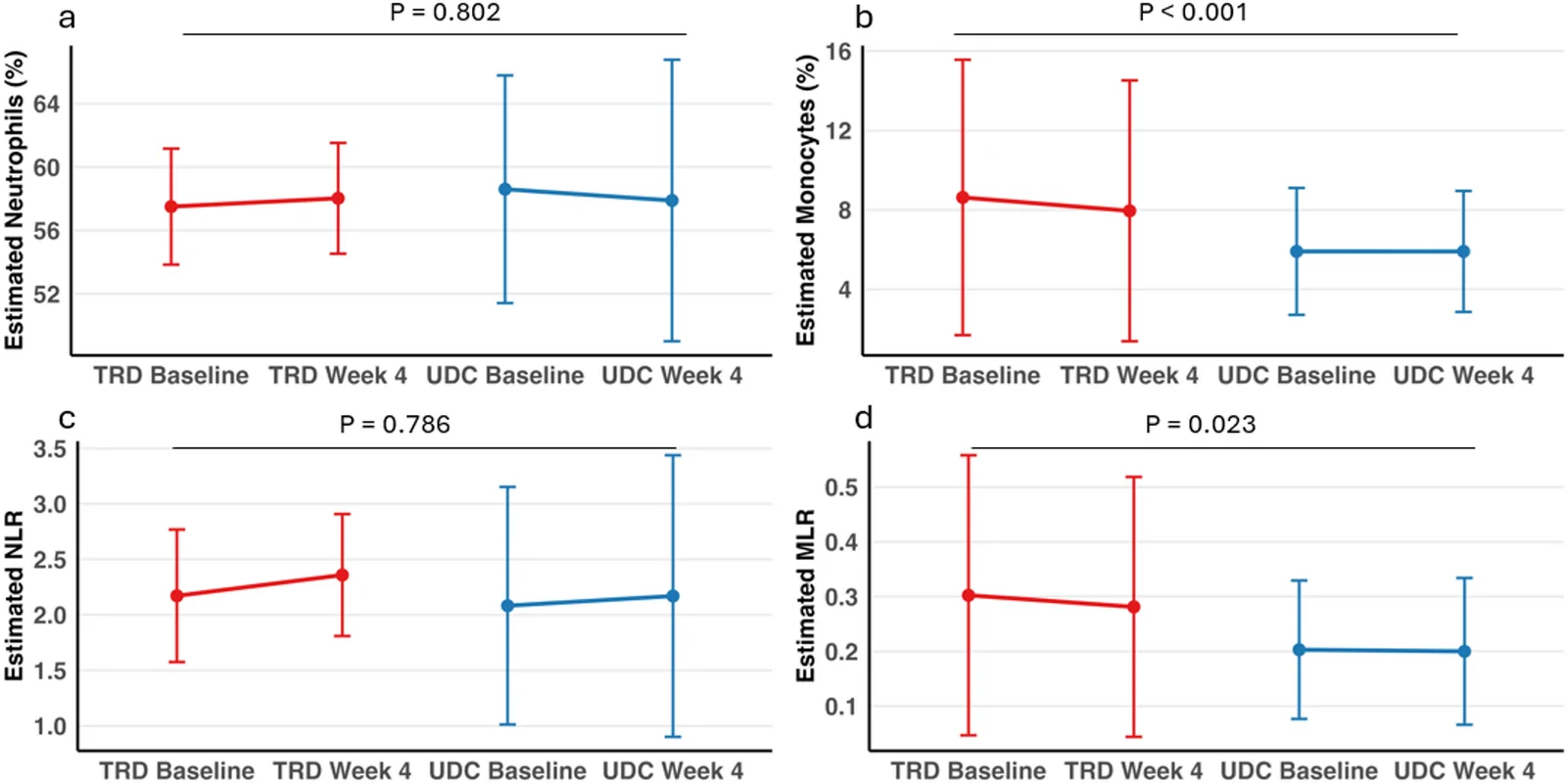
Group differences in peripheral inflammatory markers over time (TRD vs. UDC). Estimated marginal means (± 95% confidence intervals) derived from the linear mixed models are shown for TRD and UDC at baseline and week 4 (TRD = red, UDC = blue). Panels: **a** Estimated neutrophils (%), **b** Estimated monocytes (%), **c** Estimated neutrophil-to-lymphocyte ratio (NLR), **d** Estimated monocyte-to-lymphocyte ratio (MLR). P-values indicate the main effect of group (TRD vs. UDC) in the linear mixed models (averaged across timepoints): **a** *p* = 0.802, **b** *p* < 0.001, **c** *p* = 0.786, **d** *p* = 0.023

At week 4, neutrophil (%), lymphocyte (%), eosinophil (%), and basophil (%) levels again did not differ between TRD and UDC (neutrophils: *p* = 0.977; lymphocytes: *p* = 0.668; eosinophils: *p* = 0.794; basophils: *p* = 0.955). Monocyte (%) levels were higher in TRD compared with UDC (*p* = 0.004; estimated marginal means: 8.29 vs. 5.91; Fig. 2b) and lower in patients receiving ECT compared with non-ECT patients (*p* < 0.001; estimated marginal means: 6.28 vs. 7.91; Fig. 1b). Regarding treatment response, responders and non-responders did not differ overall when averaged across factors (*p* = 0.106); however, within the ECT group, responders showed lower monocyte (%) levels than non-responders (*p* = 0.025; Fig. 1b). Moreover, among responders, monocyte (%) levels were lower in patients receiving ECT compared with non-ECT patients (*p* < 0.001; estimated marginal means: 5.24 vs. 7.74). A Spearman rank correlation analysis revealed no significant associations between MADRS or BDI-II scores and cell count percentages when considering values at baseline or week 4 (all *p* > 0.05).

### Peripheral blood cell ratios

NLR did not differ between TRD and UDC (main effect of group: *p* = 0.786). However, NLR was higher in patients receiving ECT compared with non-ECT patients (main effect of ECT: *p* = 0.011; estimated marginal means: 2.60 vs. 1.79; Fig. 1c). As a confirmatory step, the model for NLR was repeated after excluding patients receiving lithium to verify that the findings were not driven by lithium use (main effect of ECT: *p* = 0.013; estimated marginal means: 2.35 vs. 1.77).

**Fig. 3:**
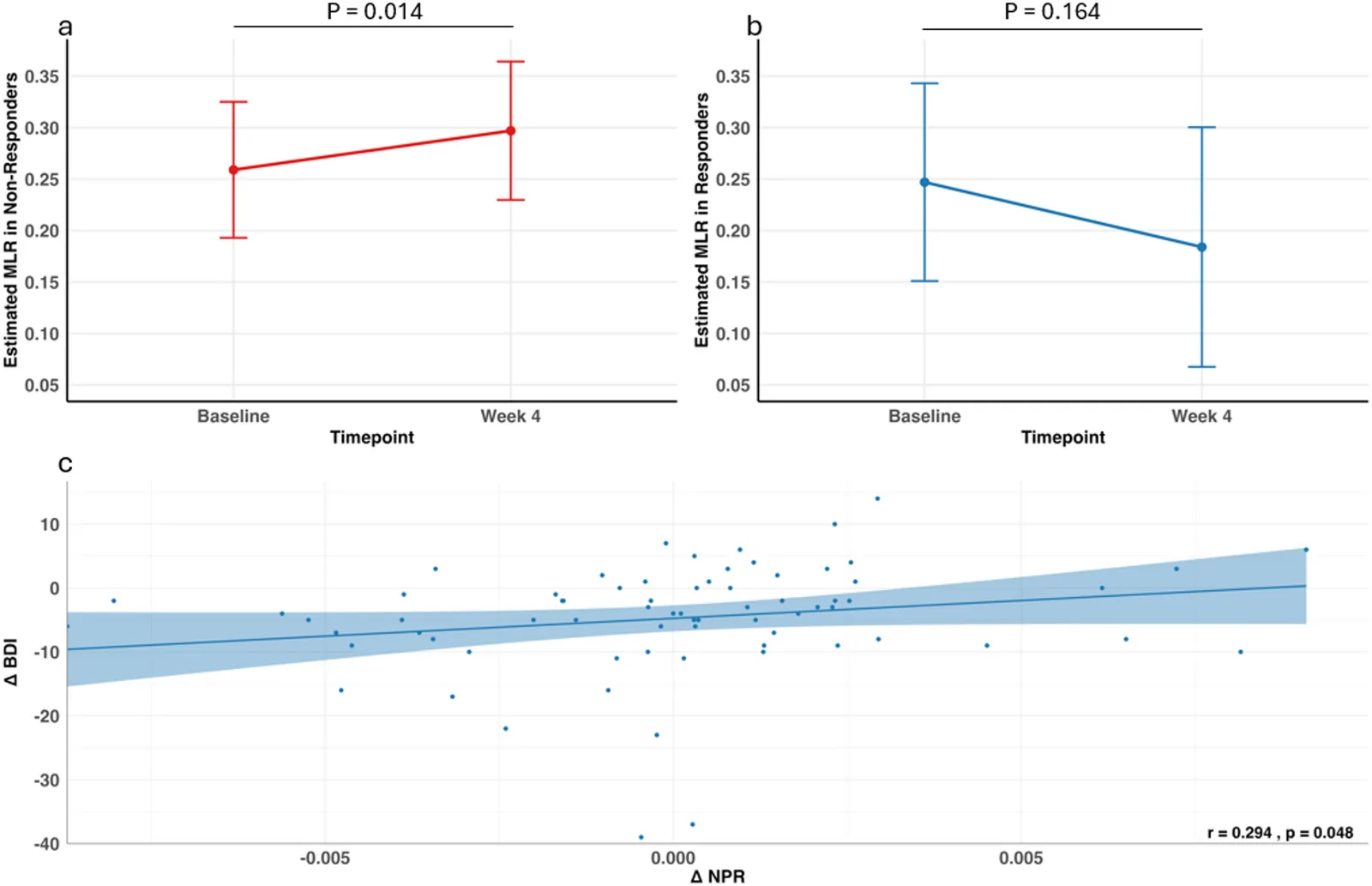
Response-related trajectories and NPR–symptom change association. **a** Estimated monocyte-to-lymphocyte ratio (MLR) over time in non-responders (baseline vs. week 4; *p* = 0.014). **b** Estimated MLR over time in responders (baseline vs. week 4; *p* = 0.164). Points represent estimated marginal means with 95% confidence intervals. **c** Association between change in neutrophil-to-platelet ratio (ΔNPR) and change in depressive symptoms (ΔBDI-II); the fitted regression line with 95% confidence band is shown (*r* = 0.294, FDR-adjusted *p* = 0.048)

MLR was higher in TRD compared with UDC (main effect of group: *p* = 0.023; estimated marginal means: 0.29 vs. 0.20; Fig. 2d). Over time, MLR changes depended on treatment response (response × timepoint interaction: *p* = 0.032). In model-derived contrasts, MLR increased from baseline to week 4 in non-responders (*p* = 0.014; estimated marginal means: 0.26 vs. 0.30; Fig. 3a), whereas no significant change was observed in responders (*p* = 0.164; Fig. 3b). At week 4, responders showed lower MLR levels than non-responders (*p* = 0.038; estimated marginal means: 0.18 vs. 0.30). NPR did not differ by group, timepoint, ECT exposure, or treatment response in the mixed models (all *p* > 0.05).

In Spearman correlation analyses with FDR correction, changes in NPR were positively correlated with changes in BDI-II scores (*r* = 0.294, FDR-adjusted *p* = 0.048; Fig. 3c), whereas changes in MLR (*r* = − 0.005, FDR-adjusted *p* = 0.982) and NLR (*r* = 0.029, FDR-adjusted *p* = 0.877) were not associated with changes in BDI-II.

### IL-6 & CRP

At baseline, neither IL-6 nor CRP differed between TRD and UDC or between TRD-ECT and non-ECT patients. In Spearman correlation analyses with FDR correction, CRP was positively correlated with IL-6 (*r* = 0.366, FDR-adjusted *p* < 0.001), total leukocytes (*r* = 0.272, FDR-adjusted *p* < 0.001), NPR (*r* = 0.266, FDR-adjusted *p* = 0.005), total neutrophils (*r* = 0.333, FDR-adjusted *p* < 0.001), neutrophils (%) (*r* = 0.199, FDR-adjusted *p* = 0.035), and total monocytes (*r* = 0.202, FDR-adjusted *p* = 0.030). No significant correlations were found between baseline IL-6 and components of the differential blood count.

At week 4, CRP levels did not differ between TRD and UDC or between TRD-ECT and non-ECT patients. Regarding treatment response, no significant differences were observed in baseline IL-6 or CRP between responders and non-responders (all *p* > 0.05).

## Discussion

We detected that monocyte levels were significantly higher in treatment-resistant patients compared to UDC patients (Fig. 2b), as well as a negative association between monocyte levels and treatment response was observed among patients treated with ECT.

Although numerous studies have focused on neuroinflammatory mechanisms in depression, data on differences in peripheral blood cell profiles between treatment-resistant and non-treatment-resistant depression remain limited. Previous studies have compared patients experiencing their first depressive episode to those with recurrent episodes (Arabska et al. 2018; Singh et al. 2022); however, even in these cases, it remains unclear whether patients with multiple episodes met the criteria for TRD. Our study tries to address this gap by examining peripheral blood cell ratios in TRD and non-TRD patients, considering MLR as a marker of chronic inflammation and NLR as a marker of acute inflammation.

To date, evidence on changes in absolute monocyte counts following ECT in (treatment-resistant) depression remains limited. However, a recent retrospective study across severe psychiatric disorders (including depressive disorders) reported significant post-ECT reductions in monocyte-related composite indices (e.g., monocyte-to-lymphocyte ratio and systemic inflammation response index), with larger decreases in bipolar mania and schizophrenia than in depressive disorders (Selçuk and Taşkın Koca 2025). Monocytes are key components of the innate immune system and play a crucial role in both infectious and autoimmune disorders. Their infiltration into inflamed tissues has been implicated in diseases such as atherosclerosis (Moore et al. 2013), Crohn’s disease (Mitsialis et al. 2020), and multiple sclerosis (Kouwenhoven et al. 2001). Elevated peripheral monocyte levels can result from increased bone marrow production (Geissmann et al. 2010), prolonged survival, or enhanced mobilization in response to inflammatory cytokines such as monocyte chemoattractant protein-1 (MCP-1) (Deshmane et al. 2009). Moreover, circulating monocytes can differentiate into macrophages or dendritic cells within tissues, and thus, perpetuate chronic inflammation and tissue remodeling (Italiani and Boraschi 2014). A previous meta-analysis demonstrated increased monocyte counts in depressed patients compared to healthy controls (Sørensen et al. 2023), which is in line with our findings and suggest that monocyte proportions differ between depressed subjects as well. In our study, TRD patients exhibited significantly higher monocyte levels than UDC patients, regardless of time point (baseline vs. four weeks). Within TRD, TRD-ECT showed lower monocyte levels than TRD-M patients. Across all groups, treatment responders had lower monocyte counts than non-responders. Interestingly, our findings contrast with previous research from our group, which reported higher levels of specific monocyte subtypes (CD11b⁺, CD91⁺, and CD14⁺⁺CD16⁺) in ECT remitters compared to non-remitters (Moschny et al. 2020a). However, that study was based on a substantially smaller sample (*n* = 21) and included patients who were not part of the present cohort. In contrast, in our current sample, lower absolute monocyte counts were associated with a better treatment response. This discrepancy may reflect differences in methodology and biological focus: while earlier work quantified activated monocyte subsets using flow cytometry, our study relies on routine hematological parameters. Selective activation or redistribution of monocyte subpopulations may occur without necessarily increasing the total monocyte count. Taken together, these observations suggest that monocyte-related immune processes may be involved in the mechanisms underlying ECT efficacy, though their exact role remains to be clarified.

Another key function of monocytes is the secretion of proinflammatory cytokines such as TNF-α and IL-6 (Dale et al. 2008). These cytokines can signal across the blood–CSF barrier and influence central nervous system function (Banks et al. 1995). Within the CNS, they may increase indoleamine 2,3-dioxygenase (IDO) activity, thereby shifting tryptophan metabolism toward the kynurenine pathway and reducing its availability for central serotonin synthesis (Müller 2017). Elevated levels of the neurotoxic kynurenine metabolite quinolinic acid (QUIN) have been reported in major depression (Savitz et al. 2015). Against this background, lower monocyte levels in ECT responders may reflect an anti-inflammatory effect that attenuates this pathway and contributes to symptom improvement.

Beyond monocytes, we also analyzed MLR. Apart from a single study examining MLR following ECT in schizophrenia (Wang et al. 2024), no prior research has investigated MLR changes related to ECT in TRD. However, a recent study in a mixed cohort of severe psychiatric disorders reported post-ECT reductions in monocyte-related composite indices, including MLR (Selçuk and Taşkın Koca 2025). MLR is a marker of chronic inflammation and has been described to be elevated in conditions such as chronic rhinosinusitis (Zagólski et al. 2018) and multiple sclerosis (Akaishi et al. 2021). Similarly, MLR levels were reportedly increased in depressed individuals compared to healthy controls (Cheng et al. 2022). Consistent with this finding, we discovered that TRD patients had higher MLR than UDC patients. Furthermore, in line with another previous study linking elevated MLR to greater symptom severity and suicidality (Puangsri and Ninla-Aesong 2021), in our study, non-responders showed a higher MLR than responders after four weeks, regardless of TRD status or treatment modality. These results could imply persistent or worsening neuroinflammation in depressed individuals not responding to treatment. Fittingly, responders to treatment showed a trend toward decreasing MLR over time; however, this result was not statistically significant. Future studies with larger sample sizes should further investigate the dynamics in MLR changes between responders and non-responders, particularly concerning remission status.

In our study, we found no differences in NLR between TRD and UDC. However, TRD-ECT patients exhibited higher NLR levels compared to those receiving antidepressant treatment alone (TRD-M and UDC; Fig. 1c). Unlike MLR, which reflects chronic inflammation, NLR is considered a marker of acute inflammation (Walzik et al. 2021), although its role in depression remains controversial. Some studies report elevated NLR in depressed patients compared to healthy controls (Cheng et al. 2022), whereas others find no significant difference (Singh et al. 2022). One study observed higher NLR in patients experiencing their first depressive episode compared to healthy controls, while patients with recurrent episodes showed lower NLR levels (Arabska et al. 2018). The authors attributed this to the anti-inflammatory effects of antidepressants. However, this explanation does not account for our findings, as patients undergoing ECT also continued antidepressant medication (Wang et al. 2019). In contrast to another study suggesting that ECT does not affect NLR (Canan et al. 2015), our results indicate increased NLR in TRD-ECT patients. Notably, the small sample size (*n* = 10) in that study may have influenced their findings (Canan et al. 2015). The observed increase in NLR in our cohort may reflect a temporary activation of the innate immune system, as ECT has been shown to elevate neutrophil counts and reduce lymphocyte levels. This short-term immune shift may contribute to its therapeutic effects.

Of note, although NLR and MLR are nonspecific markers, they have been extensively studied in neurological and rheumatic diseases with established inflammatory etiologies. In multiple sclerosis, NLR and MLR are independently associated with neurological disability and brain atrophy (Hemond et al. 2019), and NLR has been proposed as a biomarker of disease activity (Demirci et al. 2016). In autoimmune encephalitis, both NLR and MLR are associated with disease severity (Liu et al. 2022) and NLR predicts treatment failure (Broadley et al. 2020). Similarly, in systemic autoimmune rheumatic diseases, NLR and MLR are significantly elevated and correlate with inflammatory activity across conditions including rheumatoid arthritis and systemic lupus erythematosus (Yang et al. 2017). While these markers do not replace disease-specific biomarkers, their accessibility, low cost, and demonstrated associations with disease activity across multiple inflammatory conditions support their exploratory use in psychiatric populations.

It should also be considered that patients in the TRD-ECT group received general anesthesia three times weekly. Available evidence suggests that anesthetic agents such as propofol, methohexital, remifentanil, and succinylcholine primarily affect leukocyte function (e.g., chemotaxis, phagocytosis) rather than leukocyte counts at clinically relevant concentrations (Davidson et al. 1995; Yang et al. 2013). Although general anesthesia can transiently reduce circulating immune cell populations within minutes of induction (Liu et al. 2011), these changes are short-lived and attributed to redistribution rather than altered cell production. In our study, blood samples at week 4 were collected in a fasting state between 08:00 and 09:00 a.m. and not immediately after an ECT session, minimizing acute anesthesia-related effects. Nevertheless, cumulative effects of repeated anesthesia exposure cannot be fully excluded.

Overall, TRD-ECT patients showed lower MLR and monocyte levels, which may reflect reduced chronic inflammation. At the same time, they exhibited elevated NLR, suggesting a more acute inflammatory response. Together with the pronounced reduction in MADRS scores and the highest response rates, this pattern may indicate that ECT shifts TRD from a chronic inflammatory state, characterized by elevated monocytes and MLR, toward a more acute inflammatory response, reflected by elevated neutrophils and NLR. The hypothesis proposes that ECT triggers a transient, intense activation of the innate immune system (Hestad et al. 2003; Lehtimäki et al. 2008; van Buel et al. 2015). Rather than representing a harmful inflammatory reaction, this response may be beneficial and possibly therapeutic (van Buel et al. 2015). Preclinical and clinical findings suggest that this temporary immune activation could be linked to the expression and signaling of neurotrophic factors, particularly brain-derived neurotrophic factor (BDNF) (van Buel et al. 2015). BDNF is crucial for neuroplasticity and may contribute to the understanding of the antidepressant effects of ECT (Duman and Monteggia 2006; van Buel et al. 2015). Clinical studies reported that acute ECT can increase inflammatory mediators and render peripheral blood monocytes more responsive to stimulation (e.g., lipopolysaccharide), reflected by enhanced cytokine release (TNF-α, IL-6) (Hestad et al. 2003; Lehtimäki et al. 2008; Fluitman et al. 2011), and cytokines such as IL-6 and TNF-α can stimulate human monocytes to secrete neurotrophic factors including BDNF (Schulte-Herbrüggen et al. 2005). These findings indicate that, under controlled and limited conditions, immune activation may support tissue repair, synaptic remodeling, and functional recovery (Schwartz et al. 2013; Correale 2014; van Buel et al. 2015). As such, changes in immune cell composition and neurotrophin activity following ECT could reflect a more complex explanatory mechanism contributing to clinical improvement in patients with treatment-resistant depression (van Buel et al. 2015).

In addition, we examined the neutrophil-to-platelet ratio (NPR) as a potential marker of systemic inflammation. Compared to traditional inflammatory markers, composite ratios such as NPR may better capture subtle shifts in immune cell dynamics by integrating changes across different cell populations (Soehnlein 2018; Foy et al. 2022, 2025). While NPR has been less frequently investigated in psychiatric populations (Geng et al. 2024), it has been proposed as a sensitive indicator of inflammatory activation in other medical conditions (Yamamoto-Furusho and Mendieta-Escalante 2020; Zhu et al. 2025; Nguyen et al. 2026). In our cohort, NPR changes were positively correlated with changes in depressive symptom severity (ΔBDI-II; Fig. 3c), suggesting that this marker may reflect treatment-related immunological adaptations. However, given the exploratory nature of this analysis, further studies are needed to clarify its potential role in depression and ECT response.

Lastly, regarding CRP, we did not observe any association between CRP levels and symptom severity, consistent with findings from the large Netherlands Study of Depression and Anxiety (NESDA) (Lamers et al. 2019). NESDA also reported a statistically significant association between IL-6 levels and depression diagnosis (Cohen’s d ≈ 0.06) as well as depression severity (standardized β ≈ 0.03). However, the effect sizes were relatively small, indicating rather modest relationships. This could explain why we did not detect similar associations, particularly given differences in sample characteristics and size. Similarly, the positive correlation between CRP and depression severity reported by Köhler-Forsberg et al. (2017) was not confirmed in our cohort. That association was observed specifically among female participants in a clinical sample of 231 patients with major depression from the multicentre GENDEP study, whereas our study was neither powered nor designed for sex-stratified CRP analyses (Köhler-Forsberg et al. 2017).

It should be acknowledged that peripheral inflammatory markers represent an indirect measure of central neuroinflammatory processes, and the blood–brain barrier constitutes a significant barrier to direct inference. However, recent evidence supports a meaningful peripheral–central link. Felger et al. (2020) demonstrated a strong correlation between plasma and cerebrospinal fluid (CSF) CRP levels (*r* = 0.855) in unmedicated patients with major depressive disorder, with CSF CRP further correlating with depressive symptom severity (Felger et al. 2020). Meta-analyses confirmed elevated CSF levels of IL-6, TNF-α, and total protein — a marker of blood–brain barrier permeability — in depression (Enache et al. 2019; Mousten et al. 2022). Moreover, accessible peripheral measures such as IL-6 and CRP have been highlighted as reflecting neuroinflammatory processes in psychiatric disorders (Upthegrove et al. 2025). Our study is positioned as hypothesis-generating, aiming to identify cost-effective, clinically accessible markers that may complement future CSF-based investigations.

### Strengths and limitations

Our linear mixed models considered key potential confounders, including age, BMI, smoking status, and sex, enhancing the robustness of our analyses. The inclusion of follow-up measurements allowed us to capture dynamic changes in differential blood counts and related ratios over time. This is the first study to examine NPR as a peripheral inflammatory marker in treatment-resistant depression across different therapeutic regimens and at two distinct time points. Nevertheless, several limitations should be acknowledged. First, IL-6 was measured at baseline only, and lymphocyte subtypes were not available, limiting a more detailed immunophenotyping. Second, the UDC group was relatively small compared with the other groups and differed from the TRD groups in age, which may have affected group comparisons despite statistical adjustment. Third, patients with TRD represent a multimorbid and clinically complex population, and medication regimens differed substantially between the TRD groups; therefore, residual confounding by comorbidities and concomitant medication cannot be fully excluded, even though we attempted to reduce this via exclusion criteria. Somatic and psychiatric comorbidities were systematically recorded and are reported in Supplementary Table S14. However, incorporating all comorbidity categories as additional covariates was not feasible given the sample size and the number of parameters already included in the models. Fourth, the length of hospital stay and the duration of medication-only treatment prior to ECT initiation were not systematically recorded in the NEKTOR registry; these variables may have influenced inflammatory marker trajectories. Fifth, physical activity levels and participation in complementary therapies such as exercise or physiotherapy were not systematically recorded in our study. As physical activity is known to influence leukocyte counts, particularly neutrophils (Johannsen et al. 2012), this represents an additional source of unmeasured confounding. Furthermore, our study relied exclusively on peripheral blood markers, which represent an indirect measure of central neuroinflammatory processes. Although recent evidence supports a correlation between peripheral and CSF inflammatory markers in depression (Felger et al. 2020), future studies incorporating paired CSF–blood sampling are needed to validate these findings. Efforts such as the CAP (Cerebrospinal Fluid Analysis in Psychiatry) Consortium aim to address this gap; however, repeated CSF sampling remains a significant practical challenge in routine psychiatric settings. Future prospective studies should address these limitations and further evaluate inflammatory ratios in the context of symptom-based stratification, including proposed immuno-metabolic subtypes of depression (Penninx et al. 2025).

## Conclusion

Our findings suggest that treatment-resistant depression is associated with elevated markers of chronic inflammation, particularly monocyte count and MLR, and that treatment with ECT may be accompanied by a shift toward acute immune activation, as reflected by increased NLR. Lower monocyte levels and MLR, together with higher NLR, were associated with better treatment response. These parameters may represent peripheral biomarker candidates for clinical improvement in TRD. If confirmed in larger cohorts, reductions in MLR and increases in NLR during ECT treatment could help predict treatment response and support more personalized therapeutic approaches. Exploratory analyses further suggested that changes in NPR may be related to symptom change; however, this finding requires replication in larger, independent samples.

## Supplementary Information

Below is the link to the electronic supplementary material.

Supplementary Material 1

## Data Availability

Study data can be made available upon reasonable request to the corresponding author.

## Abbreviations

TRD: Treatment-resistant depression
UDC: Unmedicated depressive controls
TRD-M: Treatment-resistant depression (medication only)
TRD-ECT: Treatment-resistant depression (electroconvulsive therapy)
ECT: Electroconvulsive therapy
MLR: Monocyte-to-lymphocyte ratio
NLR: Neutrophil-to-lymphocyte ratio
NPR: Neutrophil-to-platelet ratio
CRP: C-reactive protein
IL-6: Interleukin 6
MADRS: Montgomery-åsberg depression rating scale
BDI-II: Beck depression inventory-II
FDR: False discovery rate
ANOVA: Analysis of variance
ICD-10: International classification of diseases, 10th revision
WBC: White blood cells

## Declarations

### **Declaration of AI**

Declaration of generative AI and AI-assisted technologies in the writing process: During the preparation of this work, the authors used ChatGPT (OpenAI) to improve language clarity and refine sentence structure. After using this tool, the author reviewed and edited the content as needed and takes full responsibility for the content of the publication.
