# Supplementary material for: Changes in leukocytes and CRP in different treatments of major depression
Source: J Neural Transm (Vienna). 2026 Jul 2;133(9):2521–34. doi: 10.1007/s00702-026-03187-z (PMC13687188; doi:10.1007/s00702-026-03187-z)
Supplement: Supplementary file 1 [file 702_2026_3187_MOESM1_ESM.pdf]

Supplementary Information

Article title: Changes in leukocytes and CRP in different treatments of major depression
Journal: Journal of Neural Transmission
Authors: Ammar Abo Hwach, Seda Nur Türker, Franziska Huster, Anastasia Gaspert, Alborz Bastami, Joana Wrasse, Stefan Bleich, Helge Frieling, Thomas-Nickl Jockschat, Hannah Benedictine Maier, Alexandra Neyazi
Corresponding author: Alexandra Neyazi
Department of Psychiatry and Psychotherapy, Otto von Guericke University, Magdeburg, Germany
Email: alexandra.neyazi@med.ovgu.de

1. Supplementary Results: Neutrophils (%)

1.1. Type III ANOVA (Satterthwaite)

Term	Sum Sq	Mean Sq	Num DF	Den DF	F value	p_value
Response	5.75500769	5.75500769	1	121.82149	0.216992702	0.64217272
Cohort	1.68141047	1.68141047	1	117.89105	0.063397622	0.80164269
Timepoint	0.03493872	0.03493872	1	90.62156	0.001317365	0.97112655
ECT	160.97718623	160.97718623	1	105.85891	6.069648631	0.01536316
Response:Cohort	17.82451024	17.82451024	1	115.43017	0.672073582	0.41401916
Response:Timepoint	86.07700401	86.07700401	1	90.56023	3.245535481	0.07494616
Cohort:Timepoint	2.22325765	2.22325765	1	89.19147	0.083827982	0.77284732
Response:ECT	57.67837692	57.67837692	1	102.22674	2.174764572	0.14336413
Timepoint:ECT	13.15577093	13.15577093	1	89.33702	0.496038656	0.48307780
Response:Cohort:Timepoint	33.12690870	33.12690870	1	89.22982	1.249050879	0.26673394
Response:Timepoint:ECT	81.47354572	81.47354572	1	89.23827	3.071961977	0.08308778

1.2. Main effects (overall): Estimated Marginal Means (EMMs)

1.2.1. EMMs: Cohort (overall)

Cohort	EMM	SE	df	CI_low	CI_high
TRD	59.53135	2.881836	1.160594	32.96177	86.10093
UDC	60.36276	4.441045	5.031955	48.96846	71.75706

1.2.2. Pairwise Contrasts (Tukey): Cohort (overall)

contrast	estimate	SE	df	t_ratio	p_value
TRD - UDK	-0.8314098	3.795283	123.3939	-0.219064	0.8269622

1.2.3. EMMs: ECT (overall)

ECT	EMM	SE	df	CI_low	CI_high
No ECT	57.49362	3.301663	1.785016	41.51532	73.47191
ECT	62.40050	3.475972	2.287166	49.10749	75.69351

1.2.4. Pairwise Contrasts (Tukey): ECT (overall)

contrast	estimate	SE	df	t_ratio	p_value
NO - YES	-4.906878	2.0773	113.1648	-2.362143	0.01987766

1.2.5. EMMs: Response (overall)

Response	EMM	SE	df	CI_low	CI_high
Non-responder	59.21992	2.941595	1.248659	35.55982	82.88001
Responder	60.67420	4.329779	4.532289	49.18965	72.15875

1.2.6. Pairwise Contrasts (Tukey): Response (overall)

contrast	estimate	SE	df	t_ratio	p_value
NO - YES	-1.454281	3.626071	118.4967	-0.4010625	0.6890964

1.2.7. EMMs: Timepoint (overall)

Timepoint	EMM	SE	df	CI_low	CI_high
Baseline	59.91233	3.203726	1.684345	43.33069	76.49397
Week 4	59.98178	3.531206	2.338610	46.71333	73.25023

1.2.8. Pairwise Contrasts (Tukey): Timepoint (overall)

contrast	estimate	SE	df	t_ratio	p_value
Baseline - T4	-0.06944494	1.953205	98.60791	-0.03555436	0.9717096

1.3. Estimated Marginal Means (EMMs): Cohort by Timepoint

Cohort	Timepoint	EMM	SE	df	CI_low	CI_high
TRD	Baseline	59.20856	2.912865	1.221475	34.82987	83.58724
UDC	Baseline	60.61611	4.421241	5.398543	49.49873	71.73349
TRD	Week 4	59.85415	2.972455	1.315253	37.99082	81.71747
UDC	Week 4	60.10941	5.204146	9.666956	48.45947	71.75935

1.3.1. Pairwise Contrasts (Tukey): Cohort by Timepoint

contrast	Timepoint	estimate	SE	df	t_ratio	p_value
TRD - UDK	Baseline	-1.407558	3.874128	148.6481	-0.3633224	0.7168802
TRD - UDK	Week 4	-0.255262	4.686106	184.9660	-0.0544721	0.9566178

1.4. Estimated Marginal Means (EMMs): ECT by Timepoint

ECT	Timepoint	EMM	SE	df	CI_low	CI_high
No ECT	Baseline	57.87233	3.311694	1.904615	42.91709	72.82757

ECT	Timepoint	EMM	SE	df	CI_low	CI_high
ECT	Baseline	61.95233	3.492840	2.435416	49.22653	74.67814
No ECT	Week 4	57.11490	3.590324	2.461841	44.13728	70.09253
ECT	Week 4	62.84866	3.888781	3.577888	51.53026	74.16705

1.4.1. Pairwise Contrasts (Tukey): ECT by Timepoint

contrast	Timepoint	estimate	SE	df	t_ratio	p_value
NO - YES	Baseline	-4.080001	2.297619	157.4534	-1.775752	0.07770538
NO - YES	Week 4	-5.733754	2.479600	172.5219	-2.312371	0.02193834

1.5. Estimated Marginal Means (EMMs): Response by Timepoint

Response	Timepoint	EMM	SE	df	CI_low	CI_high
Non-responder	Baseline	57.46187	2.962069	1.308307	35.48153	79.44221
Responder	Baseline	62.36280	4.260996	4.675429	51.17685	73.54874
Non-responder	Week 4	60.97796	3.053599	1.440928	41.51264	80.44328
Responder	Week 4	58.98560	5.114967	8.961085	47.40707	70.56412

1.5.1. Pairwise Contrasts (Tukey): Response by Timepoint

contrast	Timepoint	estimate	SE	df	t_ratio	p_value
NO - YES	Baseline	-4.900922	3.578329	140.0125	-1.3696122	0.1730006
NO - YES	Week 4	1.992361	4.593152	175.7593	0.4337677	0.6649891

1.6. Estimated Marginal Means (EMMs): Timepoint by Response

Timepoint	Response	EMM	SE	df	CI_low	CI_high
Baseline	Non-responder	57.46187	2.962069	1.308307	35.48153	79.44221
Week 4	Non-responder	60.97796	3.053599	1.440928	41.51264	80.44328
Baseline	Responder	62.36280	4.260996	4.675429	51.17685	73.54874
Week 4	Responder	58.98560	5.114967	8.961085	47.40707	70.56412

1.6.1. Pairwise Contrasts (Tukey): Timepoint by Response

contrast	Response	estimate	SE	df	t_ratio	p_value
Baseline - T4	Non-responder	-3.516087	1.258856	94.77051	-2.7930801	0.006316857
Baseline - T4	Responder	3.377197	3.694591	99.05225	0.9140923	0.362888121

1.7. Estimated Marginal Means (EMMs): Response by ECT

Response	ECT	EMM	SE	df	CI_low	CI_high
Non-responder	No ECT	58.22032	2.862508	1.139624	30.85940	85.58124
Responder	No ECT	56.76691	4.525195	5.540513	45.46771	68.06612
Non-responder	ECT	60.21951	3.363315	2.039410	46.01304	74.42598
Responder	ECT	64.58148	4.843453	7.387145	53.24910	75.91387

1.7.1. Pairwise Contrasts (Tukey): Response by ECT

contrast	ECT	estimate	SE	df	t_ratio	p_value
NO - YES	No ECT	1.453409	3.706587	124.8484	0.3921152	0.6956417
NO - YES	ECT	-4.361971	4.605681	105.6535	-0.9470848	0.3457567

1.8. Estimated Marginal Means (EMMs): ECT by Response

ECT	Response	EMM	SE	df	CI_low	CI_high
No ECT	Non-responder	58.22032	2.862508	1.139624	30.85940	85.58124
ECT	Non-responder	60.21951	3.363315	2.039410	46.01304	74.42598
No ECT	Responder	56.76691	4.525195	5.540513	45.46771	68.06612
ECT	Responder	64.58148	4.843453	7.387145	53.24910	75.91387

1.8.1. Pairwise Contrasts (Tukey): ECT by Response

contrast	Response	estimate	SE	df	t_ratio	p_value
NO - YES	Non-responder	-1.999188	2.097559	93.2637	-0.9531023	0.34300198
NO - YES	Responder	-7.814568	3.589555	115.3838	-2.1770299	0.03151494

2. Results: Neutrophils (%) - Baseline

2.1. Type III ANOVA (Satterthwaite)

Term	Sum Sq	Mean Sq	Num DF	Den DF	F value	p_value
Response	56.07106	56.07106	1	73.72349	2.0445906	0.15697243
Cohort	14.62956	14.62956	1	74.88785	0.5334562	0.46743623
ECT	97.96300	97.96300	1	76.17990	3.5721496	0.06256015
Response:Cohort	123.18550	123.18550	1	74.60666	4.4918695	0.03738360
Response:ECT	130.40534	130.40534	1	73.66546	4.7551358	0.03240471

2.2. Main effects (overall): Estimated Marginal Means (EMMs)

2.2.1. EMMs: Cohort

Cohort	EMM	SE	df	CI_low	CI_high
TRD	58.38451	2.748223	1.313126	38.11595	78.65307
UDC	61.03104	6.120458	8.217397	46.98197	75.08011

2.2.2. Pairwise Contrasts (Tukey): Cohort

contrast	estimate	SE	df	t_ratio	p_value
TRD - UDK	-2.646531	5.687945	67.76514	-0.4652878	0.6432172

2.2.3. EMMs: ECT

ECT	EMM	SE	df	CI_low	CI_high
No ECT	57.76144	3.848667	2.336910	43.29192	72.23095

ECT	EMM	SE	df	CI_low	CI_high
ECT	61.65411	4.046789	3.226861	49.27279	74.03544

2.2.4. Pairwise Contrasts (Tukey): ECT

contrast	estimate	SE	df	t_ratio	p_value
NO - YES	-3.892675	2.169039	75.08784	-1.794655	0.07673336

2.2.5. EMMs: Response

Response	EMM	SE	df	CI_low	CI_high
Non-responder	57.20380	2.772290	1.355610	37.79359	76.61402
Responder	62.21175	6.113112	7.722381	48.02619	76.39731

2.2.6. Pairwise Contrasts (Tukey): Response

contrast	estimate	SE	df	t_ratio	p_value
NO - YES	-5.007946	5.695498	43.48033	-0.8792816	0.3840824

2.3. Estimated Marginal Means (EMMs): Response by ECT

Response	ECT	EMM	SE	df	CI_low	CI_high
Non-responder	No ECT	57.46783	2.682131	1.195562	34.10858	80.82709
Responder	No ECT	58.05505	6.221062	8.884822	43.95417	72.15592
Non-responder	ECT	56.93978	3.226579	2.411840	45.09857	68.78098
Responder	ECT	66.36845	6.581021	12.654769	52.11149	80.62541

2.3.1. Pairwise Contrasts (Tukey): Response by ECT

contrast	ECT	estimate	SE	df	t_ratio	p_value
NO - YES	No ECT	-0.5872132	5.704553	73.32170	-0.1029376	0.9182935
NO - YES	ECT	-9.4286797	6.475746	37.85207	-1.4559991	0.1536378

2.4. Estimated Marginal Means (EMMs): ECT by Response

ECT	Response	EMM	SE	df	CI_low	CI_high
No ECT	Non-responder	57.46783	2.682131	1.195562	34.10858	80.82709
ECT	Non-responder	56.93978	3.226579	2.411840	45.09857	68.78098
No ECT	Responder	58.05505	6.221062	8.884822	43.95417	72.15592
ECT	Responder	66.36845	6.581021	12.654769	52.11149	80.62541

2.4.1. Pairwise Contrasts (Tukey): ECT by Response

contrast	Response	estimate	SE	df	t_ratio	p_value
NO - YES	Non-responder	0.528058	2.113507	67.95342	0.2498492	0.80345772
NO - YES	Responder	-8.313408	3.813445	76.03383	-2.1800259	0.03235031

2.5. Estimated Marginal Means (EMMs): Response by Cohort

Response	Cohort	EMM	SE	df	CI_low	CI_high
Non-responder	TRD	59.73454	2.676045	1.268224	38.79190	80.67717
Responder	TRD	57.03449	3.225568	2.283591	44.68356	69.38541
Non-responder	UDC	54.67307	3.120738	2.016025	41.34738	67.99876
Responder	UDC	67.38902	11.313440	39.107621	44.50744	90.27060

2.5.1. Pairwise Contrasts (Tukey): Response by Cohort

contrast	Cohort	estimate	SE	df	t_ratio	p_value
NO - YES	TRD	2.700052	2.218129	70.66849	1.217266	0.2275525
NO - YES	UDC	-12.715945	11.208294	60.73840	-1.134512	0.2610375

2.6. Estimated Marginal Means (EMMs): Cohort by Response

Cohort	Response	EMM	SE	df	CI_low	CI_high
TRD	Non-responder	59.73454	2.676045	1.268224	38.79190	80.67717
UDC	Non-responder	54.67307	3.120738	2.016025	41.34738	67.99876
TRD	Responder	57.03449	3.225568	2.283591	44.68356	69.38541
UDC	Responder	67.38902	11.313440	39.107621	44.50744	90.27060

2.6.1. Pairwise Contrasts (Tukey): Cohort by Response

contrast	Response	estimate	SE	df	t_ratio	p_value
TRD - UDK	Non-responder	5.061468	1.748738	72.01579	2.8943542	0.00502482
TRD - UDK	Responder	-10.354530	11.283435	70.70850	-0.9176753	0.36190849

3. Supplementary Results: Neutrophils (%) - Week 4

3.1. Type III ANOVA (Satterthwaite)

Term	Sum Sq	Mean Sq	Num DF	Den DF	F value	p_value
Response	0.14471174691	0.14471174691	1	64.55547	39.04983106	0.00000003677409
Cohort	0.02013496302	0.02013496302	1	81.74511	5.43333158	0.02221991622060
ECT	0.02112154020	0.02112154020	1	83.47453	5.69955511	0.01922800845945
Response:Cohort	0.01960026599	0.01960026599	1	81.48214	5.28904593	0.02401859016772
Response:ECT	0.00006371959	0.00006371959	1	83.81159	0.01719445	0.89598860377008

3.2. Main effects (overall): Estimated Marginal Means (EMMs)

3.2.1. EMMs: Cohort

Cohort	EMM	SE	df	CI_low	CI_high
TRD	60.97229	4.787912	1.113292	12.98110	108.96347
UDC	54.94436	5.510971	1.410826	18.68642	91.20231

3.2.2. Pairwise Contrasts (Tukey): Cohort

contrast	estimate	SE	df	t_ratio	p_value
TRD - UDK	6.027923	3.09817	1.527139	1.94564	0.2289113

3.2.3. EMMs: ECT

ECT	EMM	SE	df	CI_low	CI_high
No ECT	54.83739	4.466536	1.197863	16.07776	93.59703

ECT	EMM	SE	df	CI_low	CI_high
ECT	61.07926	5.919984	1.480705	24.80335	97.35518

3.2.4. Pairwise Contrasts (Tukey): ECT

contrast	estimate	SE	df	t_ratio	p_value
NO - YES	-6.241871	3.605714	23.91132	-1.731105	0.09631884

3.2.5. EMMs: Response

Response	EMM	SE	df	CI_low	CI_high
Non-responder	62.57307	4.528461	1.2711268	27.27430	97.87185
Responder	53.34358	5.672879	0.8713733	-49.45355	156.14071

3.2.6. Pairwise Contrasts (Tukey): Response

contrast	estimate	SE	df	t_ratio	p_value
NO - YES	9.229497	2.895843	0.1127492	3.187154	0.7208088

3.3. Estimated Marginal Means (EMMs): Response by ECT

Response	ECT	EMM	SE	df	CI_low	CI_high
Non-responder	No ECT	59.63344	4.261394	1.160145	20.31505	98.95182
Responder	No ECT	50.04135	5.184050	1.636132	22.23799	77.84470
Non-responder	ECT	65.51271	5.292727	2.134727	44.06328	86.96215
Responder	ECT	56.64581	7.456788	1.385789	6.21809	107.07353

3.3.1. Pairwise Contrasts (Tukey): Response by ECT

contrast	ECT	estimate	SE	df	t_ratio	p_value
NO - YES	No ECT	9.592090	3.204359	1.531224	2.993450	0.1308707
NO - YES	ECT	8.866905	5.200812	2.413783	1.704908	0.2084445

3.4. Estimated Marginal Means (EMMs): ECT by Response

ECT	Response	EMM	SE	df	CI_low	CI_high
No ECT	Non-responder	59.63344	4.261394	1.160145	20.31505	98.95182
ECT	Non-responder	65.51271	5.292727	2.134727	44.06328	86.96215
No ECT	Responder	50.04135	5.184050	1.636132	22.23799	77.84470
ECT	Responder	56.64581	7.456788	1.385789	6.21809	107.07353

3.4.1. Pairwise Contrasts (Tukey): ECT by Response

contrast	Response	estimate	SE	df	t_ratio	p_value
NO - YES	Non-responder	-5.879278	3.212012	71.06042	-1.830404	0.07138305
NO - YES	Responder	-6.604463	6.019126	33.84652	-1.097246	0.28027900

3.5. Estimated Marginal Means (EMMs): Response by Cohort

Response	Cohort	EMM	SE	df	CI_low	CI_high
Non-responder	TRD	62.62299	4.143517	1.2195459	27.84399	97.40199
Responder	TRD	59.32159	6.151166	1.4444343	20.25089	98.39228
Non-responder	UDC	62.52316	5.322097	1.7607431	36.36776	88.67856
Responder	UDC	47.36557	6.619314	0.7529369	-140.43680	235.16794

3.5.1. Pairwise Contrasts (Tukey): Response by Cohort

contrast	Cohort	estimate	SE	df	t_ratio	p_value
NO - YES	TRD	3.301405	4.279574	7.7015980	0.7714331	0.4634589
NO - YES	UDC	15.157589	4.774608	0.2656329	3.1746250	0.5248576

3.6. Estimated Marginal Means (EMMs): Cohort by Response

Cohort	Response	EMM	SE	df	CI_low	CI_high
TRD	Non-responder	62.62299	4.143517	1.2195459	27.84399	97.40199
UDC	Non-responder	62.52316	5.322097	1.7607431	36.36776	88.67856
TRD	Responder	59.32159	6.151166	1.4444343	20.25089	98.39228
UDC	Responder	47.36557	6.619314	0.7529369	-140.43680	235.16794

3.6.1. Pairwise Contrasts (Tukey): Cohort by Response

contrast	Response	estimate	SE	df	t_ratio	p_value
TRD - UDK	Non-responder	0.09983093	2.993168	33.128996	0.03335294	0.9735933
TRD - UDK	Responder	11.95601524	5.880313	1.024266	2.03322766	0.2863554

4. Supplementary Results: Neutrophils (%) - Baseline

4.1. Type III ANOVA (Satterthwaite)

Term	Sum Sq	Mean Sq	Num DF	Den DF	F value	p_value
Response	56.07106	56.07106	1	73.72349	2.0445906	0.15697243
Cohort	14.62956	14.62956	1	74.88785	0.5334562	0.46743623
ECT	97.96300	97.96300	1	76.17990	3.5721496	0.06256015
Response:Cohort	123.18550	123.18550	1	74.60666	4.4918695	0.03738360
Response:ECT	130.40534	130.40534	1	73.66546	4.7551358	0.03240471

4.2. Main effects (overall): Estimated Marginal Means (EMMs)

4.2.1. EMMs: Cohort

Cohort	EMM	SE	df	CI_low	CI_high
TRD	58.38451	2.748223	1.313126	38.11595	78.65307
UDC	61.03104	6.120458	8.217397	46.98197	75.08011

4.2.2. Pairwise Contrasts (Tukey): Cohort

contrast	estimate	SE	df	t_ratio	p_value
TRD - UDK	-2.646531	5.687945	67.76514	-0.4652878	0.6432172

4.2.3. EMMs: ECT

ECT	EMM	SE	df	CI_low	CI_high
No ECT	57.76144	3.848667	2.336910	43.29192	72.23095

ECT	EMM	SE	df	CI_low	CI_high
ECT	61.65411	4.046789	3.226861	49.27279	74.03544

4.2.4. Pairwise Contrasts (Tukey): ECT

contrast	estimate	SE	df	t_ratio	p_value
NO - YES	-3.892675	2.169039	75.08784	-1.794655	0.07673336

4.2.5. EMMs: Response

Response	EMM	SE	df	CI_low	CI_high
Non-responder	57.20380	2.772290	1.355610	37.79359	76.61402
Responder	62.21175	6.113112	7.722381	48.02619	76.39731

4.2.6. Pairwise Contrasts (Tukey): Response

contrast	estimate	SE	df	t_ratio	p_value
NO - YES	-5.007946	5.695498	43.48033	-0.8792816	0.3840824

4.3. Estimated Marginal Means (EMMs): Response by ECT

Response	ECT	EMM	SE	df	CI_low	CI_high
Non-responder	No ECT	57.46783	2.682131	1.195562	34.10858	80.82709
Responder	No ECT	58.05505	6.221062	8.884822	43.95417	72.15592
Non-responder	ECT	56.93978	3.226579	2.411840	45.09857	68.78098
Responder	ECT	66.36845	6.581021	12.654769	52.11149	80.62541

4.3.1. Pairwise Contrasts (Tukey): Response by ECT

contrast	ECT	estimate	SE	df	t_ratio	p_value
NO - YES	No ECT	-0.5872132	5.704553	73.32170	-0.1029376	0.9182935
NO - YES	ECT	-9.4286797	6.475746	37.85207	-1.4559991	0.1536378

4.4. Estimated Marginal Means (EMMs): ECT by Response

ECT	Response	EMM	SE	df	CI_low	CI_high
No ECT	Non-responder	57.46783	2.682131	1.195562	34.10858	80.82709
ECT	Non-responder	56.93978	3.226579	2.411840	45.09857	68.78098
No ECT	Responder	58.05505	6.221062	8.884822	43.95417	72.15592
ECT	Responder	66.36845	6.581021	12.654769	52.11149	80.62541

4.4.1. Pairwise Contrasts (Tukey): ECT by Response

contrast	Response	estimate	SE	df	t_ratio	p_value
NO - YES	Non-responder	0.528058	2.113507	67.95342	0.2498492	0.80345772
NO - YES	Responder	-8.313408	3.813445	76.03383	-2.1800259	0.03235031

4.5. Estimated Marginal Means (EMMs): Response by Cohort

Response	Cohort	EMM	SE	df	CI_low	CI_high
Non-responder	TRD	59.73454	2.676045	1.268224	38.79190	80.67717
Responder	TRD	57.03449	3.225568	2.283591	44.68356	69.38541
Non-responder	UDC	54.67307	3.120738	2.016025	41.34738	67.99876
Responder	UDC	67.38902	11.313440	39.107621	44.50744	90.27060

4.5.1. Pairwise Contrasts (Tukey): Response by Cohort

contrast	Cohort	estimate	SE	df	t_ratio	p_value
NO - YES	TRD	2.700052	2.218129	70.66849	1.217266	0.2275525
NO - YES	UDC	-12.715945	11.208294	60.73840	-1.134512	0.2610375

4.6. Estimated Marginal Means (EMMs): Cohort by Response

Cohort	Response	EMM	SE	df	CI_low	CI_high
TRD	Non-responder	59.73454	2.676045	1.268224	38.79190	80.67717
UDC	Non-responder	54.67307	3.120738	2.016025	41.34738	67.99876
TRD	Responder	57.03449	3.225568	2.283591	44.68356	69.38541
UDC	Responder	67.38902	11.313440	39.107621	44.50744	90.27060

4.6.1. Pairwise Contrasts (Tukey): Cohort by Response

contrast	Response	estimate	SE	df	t_ratio	p_value
TRD - UDK	Non-responder	5.061468	1.748738	72.01579	2.8943542	0.00502482
TRD - UDK	Responder	-10.354530	11.283435	70.70850	-0.9176753	0.36190849

5. Supplementary Results: Lymphocytes (%)

5.1. Type III ANOVA (Satterthwaite)

Term	Sum Sq	Mean Sq	Num DF	Den DF	F value	p_value
Response	2.2614307	2.2614307	1	126.00034	0.111659822	0.73881784
Cohort	4.3443245	4.3443245	1	122.52259	0.214504252	0.64408130
Timepoint	4.4383427	4.4383427	1	93.12444	0.219146468	0.64078485
ECT	73.0114539	73.0114539	1	110.59315	3.604994801	0.06021234
Response:Cohort	0.0583691	0.0583691	1	119.98069	0.002882018	0.95727588
Response:Timepoint	78.1492790	78.1492790	1	93.12991	3.858678734	0.05247042
Cohort:Timepoint	1.0947121	1.0947121	1	92.18147	0.054052225	0.81667181
Response:ECT	18.8713671	18.8713671	1	105.79572	0.931787777	0.33660016
Timepoint:ECT	5.2042035	5.2042035	1	91.56728	0.256961413	0.61343401
Response:Cohort:Timepoint	28.3117612	28.3117612	1	92.21366	1.397914254	0.24011284
Response:Timepoint:ECT	29.1210170	29.1210170	1	91.59888	1.437871858	0.23357683

5.2. Main effects (overall): Estimated Marginal Means (EMMs)

5.2.1. EMMs: Cohort (overall)

Cohort	EMM	SE	df	CI_low	CI_high
TRD	30.19200	1.054785	2.771142	26.67279	33.71121
UDC	31.59929	3.225946	77.729160	25.17657	38.02201

5.2.2. Pairwise Contrasts (Tukey): Cohort (overall)

contrast	estimate	SE	df	t_ratio	p_value
TRD - UDK	-1.40729	3.473404	124.0446	-0.4051616	0.6860567

5.2.3. EMMs: ECT (overall)

ECT	EMM	SE	df	CI_low	CI_high
No ECT	32.63587	1.758674	15.59071	28.89968	36.37206
ECT	29.15541	2.055494	30.19438	24.95867	33.35216

5.2.4. Pairwise Contrasts (Tukey): ECT (overall)

contrast	estimate	SE	df	t_ratio	p_value
NO - YES	3.480459	1.913613	113.5883	1.818789	0.0715782

5.2.5. EMMs: Response (overall)

Response	EMM	SE	df	CI_low	CI_high
Non-responder	30.41622	1.118066	3.798636	27.24598	33.58646
Responder	31.37507	3.118600	72.337226	25.15875	37.59139

5.2.6. Pairwise Contrasts (Tukey): Response (overall)

contrast	estimate	SE	df	t_ratio	p_value
NO - YES	-0.9588484	3.313218	119.2535	-0.289401	0.7727771

5.2.7. EMMs: Timepoint (overall)

Timepoint	EMM	SE	df	CI_low	CI_high
Baseline	30.50377	1.641125	14.02099	26.98440	34.02314
Week 4	31.28751	2.060399	30.35661	27.08169	35.49334

5.2.8. Pairwise Contrasts (Tukey): Timepoint (overall)

contrast	estimate	SE	df	t_ratio	p_value
Baseline - T4	-0.7837407	1.703821	98.18514	-0.4599901	0.6465403

5.3. Estimated Marginal Means (EMMs): Cohort by Timepoint

Cohort	Timepoint	EMM	SE	df	CI_low	CI_high
TRD	Baseline	30.00322	1.144116	3.796081	26.75821	33.24824
UDC	Baseline	31.00432	3.216452	87.061193	24.61134	37.39730
TRD	Week 4	30.38077	1.204684	5.023988	27.28847	33.47307
UDC	Week 4	32.19426	3.996332	138.611364	24.29260	40.09591

5.3.1. Pairwise Contrasts (Tukey): Cohort by Timepoint

contrast	Timepoint	estimate	SE	df	t_ratio	p_value
TRD - UDK	Baseline	-1.001095	3.540618	146.3707	-0.2827458	0.7777711
TRD - UDK	Week 4	-1.813485	4.226451	185.9160	-0.4290798	0.6683619

5.4. Estimated Marginal Means (EMMs): ECT by Timepoint

ECT	Timepoint	EMM	SE	df	CI_low	CI_high
No ECT	Baseline	31.98530	1.774129	19.14773	28.27395	35.69666

ECT	Timepoint	EMM	SE	df	CI_low	CI_high
ECT	Baseline	29.02224	2.106001	35.61551	24.74947	33.29501
No ECT	Week 4	33.28644	2.148713	32.99653	28.91483	37.65805
ECT	Week 4	29.28859	2.528629	63.88251	24.23689	34.34028

5.4.1. Pairwise Contrasts (Tukey): ECT by Timepoint

contrast	Timepoint	estimate	SE	df	t_ratio	p_value
NO - YES	Baseline	2.963064	2.095801	155.4841	1.413810	0.15941672
NO - YES	Week 4	3.997854	2.245192	170.4810	1.780629	0.07675389

5.5. Estimated Marginal Means (EMMs): Response by Timepoint

Response	Timepoint	EMM	SE	df	CI_low	CI_high
Non-responder	Baseline	31.66867	1.200145	5.112033	28.60382	34.73351
Responder	Baseline	29.33888	3.050248	79.191066	23.26774	35.41002
Non-responder	Week 4	29.16377	1.289732	6.902045	26.10524	32.22230
Responder	Week 4	33.41126	3.916960	131.055463	25.66261	41.15991

5.5.1. Pairwise Contrasts (Tukey): Response by Timepoint

contrast	Timepoint	estimate	SE	df	t_ratio	p_value
NO - YES	Baseline	2.329789	3.273463	138.1221	0.7117199	0.4778386
NO - YES	Week 4	-4.247486	4.126862	176.9326	-1.0292290	0.3047770

5.6. Estimated Marginal Means (EMMs): Timepoint by Response

Timepoint	Response	EMM	SE	df	CI_low	CI_high
Baseline	Non-responder	31.66867	1.200145	5.112033	28.60382	34.73351
Week 4	Non-responder	29.16377	1.289732	6.902045	26.10524	32.22230
Baseline	Responder	29.33888	3.050248	79.191066	23.26774	35.41002
Week 4	Responder	33.41126	3.916960	131.055463	25.66261	41.15991

5.6.1. Pairwise Contrasts (Tukey): Timepoint by Response

contrast	Response	estimate	SE	df	t_ratio	p_value
Baseline - T4	Non-responder	2.504896	1.098737	93.52532	2.279796	0.0248926
Baseline - T4	Responder	-4.072378	3.223431	98.78039	-1.263367	0.2094309

5.7. Estimated Marginal Means (EMMs): Response by ECT

Response	ECT	EMM	SE	df	CI_low	CI_high
Non-responder	No ECT	31.28332	0.9809097	2.336847	27.59540	34.97124
Responder	No ECT	33.98842	3.3114611	86.058013	27.40552	40.57133
Non-responder	ECT	29.54912	1.8450300	20.478958	25.70621	33.39202
Responder	ECT	28.76171	3.7316238	94.017885	21.35250	36.17092

5.7.1. Pairwise Contrasts (Tukey): Response by ECT

contrast	ECT	estimate	SE	df	t_ratio	p_value
NO - YES	No ECT	-2.7051036	3.388832	125.2517	-0.7982407	0.4262417
NO - YES	ECT	0.7874067	4.214033	105.4854	0.1868535	0.8521344

5.8. Estimated Marginal Means (EMMs): ECT by Response

ECT	Response	EMM	SE	df	CI_low	CI_high
No ECT	Non-responder	31.28332	0.9809097	2.336847	27.59540	34.97124
ECT	Non-responder	29.54912	1.8450300	20.478958	25.70621	33.39202
No ECT	Responder	33.98842	3.3114611	86.058013	27.40552	40.57133
ECT	Responder	28.76171	3.7316238	94.017885	21.35250	36.17092

5.8.1. Pairwise Contrasts (Tukey): ECT by Response

contrast	Response	estimate	SE	df	t_ratio	p_value
NO - YES	Non-responder	1.734204	1.931929	93.48761	0.8976542	0.3716759
NO - YES	Responder	5.226715	3.298321	115.18845	1.5846593	0.1157854

6. Supplementary Results: Monocytes (%)

6.1. Type III ANOVA (Satterthwaite)

Term	Sum Sq	Mean Sq	Num DF	Den DF	F value	p_value
Response	5.90013947	5.90013947	1	75.11327	4.67978449	0.03370075724
Cohort	18.93944156	18.93944156	1	125.67100	15.02210334	0.00017022545
Timepoint	0.86021501	0.86021501	1	104.35567	0.68229249	0.41068290379
ECT	20.65118016	20.65118016	1	124.47951	16.37979458	0.00009040725
Response:Cohort	19.56270210	19.56270210	1	97.87270	15.51645181	0.00015337871
Response:Timepoint	0.30677366	0.30677366	1	104.07181	0.24332215	0.62285620573
Cohort:Timepoint	0.78113782	0.78113782	1	103.27672	0.61957123	0.43300929920
Response:ECT	5.88556543	5.88556543	1	121.09071	4.66822487	0.03269509071
Timepoint:ECT	0.13088674	0.13088674	1	103.14743	0.10381479	0.74795101373
Response:Cohort:Timepoint	0.01474079	0.01474079	1	103.29795	0.01169188	0.91410310636
Response:Timepoint:ECT	4.21977536	4.21977536	1	103.15474	3.34697839	0.07021690065

6.2. Main effects (overall): Estimated Marginal Means (EMMs)

6.2.1. EMMs: Cohort (overall)

Cohort	EMM	SE	df	CI_low	CI_high
TRD	8.288542	0.7336871	1.103968	0.8051056	15.771979
UDC	5.906206	0.9721499	2.478926	2.4098767	9.402535

6.2.2. Pairwise Contrasts (Tukey): Cohort (overall)

contrast	estimate	SE	df	t_ratio	p_value
TRD - UDK	2.382337	0.7196722	16.94341	3.310308	0.004151987

6.2.3. EMMs: ECT (overall)

ECT	EMM	SE	df	CI_low	CI_high
No ECT	7.913588	0.7886609	1.325164	2.184386	13.64279
ECT	6.281161	0.8327703	1.641806	1.834033	10.72829

6.2.4. Pairwise Contrasts (Tukey): ECT (overall)

contrast	estimate	SE	df	t_ratio	p_value
NO - YES	1.632427	0.4268121	112.1945	3.824697	0.0002156343

6.2.5. EMMs: Response (overall)

Response	EMM	SE	df	CI_low	CI_high
Non-responder	7.699461	0.7389446	1.133854	0.5639763	14.83495
Responder	6.495287	0.9516471	2.229049	2.7785890	10.21198

6.2.6. Pairwise Contrasts (Tukey): Response (overall)

contrast	estimate	SE	df	t_ratio	p_value
NO - YES	1.204175	0.6741858	9.48632	1.786117	0.1060158

6.2.7. EMMs: Timepoint (overall)

Timepoint	EMM	SE	df	CI_low	CI_high
Baseline	7.266771	0.7844611	1.349063	1.731534	12.80201
Week 4	6.927977	0.8347237	1.629026	2.426669	11.42929

6.2.8. Pairwise Contrasts (Tukey): Timepoint (overall)

contrast	estimate	SE	df	t_ratio	p_value
Baseline - T4	0.3387934	0.4188908	102.3049	0.8087871	0.4205145

6.3. Estimated Marginal Means (EMMs): Cohort by Timepoint

Cohort	Timepoint	EMM	SE	df	CI_low	CI_high
TRD	Baseline	8.626439	0.7412385	1.151503	1.686009	15.566869
UDC	Baseline	5.907103	0.9828424	2.885640	2.707950	9.106255
TRD	Week 4	7.950646	0.7481964	1.190080	1.377655	14.523637
UDC	Week 4	5.905309	1.1209492	4.217542	2.855354	8.955263

6.3.1. Pairwise Contrasts (Tukey): Cohort by Timepoint

contrast	Timepoint	estimate	SE	df	t_ratio	p_value
TRD - UDK	Baseline	2.719336	0.7545265	49.57804	3.60403	0.0007254557
TRD - UDK	Week 4	2.045337	0.9195627	26.12317	2.22425	0.0349821460

6.4. Estimated Marginal Means (EMMs): ECT by Timepoint

ECT	Timepoint	EMM	SE	df	CI_low	CI_high
No ECT	Baseline	8.123615	0.7959691	1.430292	2.993592	13.253637

ECT	Timepoint	EMM	SE	df	CI_low	CI_high
ECT	Baseline	6.409927	0.8429358	1.770349	2.292711	10.527143
No ECT	Week 4	7.703560	0.8400710	1.651178	3.248965	12.158156
ECT	Week 4	6.152394	0.9061694	2.268054	2.663397	9.641391

6.4.1. Pairwise Contrasts (Tukey): ECT by Timepoint

contrast	Timepoint	estimate	SE	df	t_ratio	p_value
NO - YES	Baseline	1.713688	0.4761281	162.0557	3.599216	0.0004237429
NO - YES	Week 4	1.551166	0.5164010	175.9765	3.003802	0.0030551023

6.5. Estimated Marginal Means (EMMs): Response by Timepoint

Response	Timepoint	EMM	SE	df	CI_low	CI_high
Non-responder	Baseline	7.768034	0.7469768	1.181674	1.116675	14.419394
Responder	Baseline	6.765507	0.9531742	2.526365	3.383545	10.147469
Non-responder	Week 4	7.630888	0.7557088	1.241411	1.488961	13.772816
Responder	Week 4	6.225066	1.1027300	3.834595	3.110603	9.339529

6.5.1. Pairwise Contrasts (Tukey): Response by Timepoint

contrast	Timepoint	estimate	SE	df	t_ratio	p_value
NO - YES	Baseline	1.002527	0.6866686	27.58872	1.459987	0.1555882
NO - YES	Week 4	1.405822	0.8872226	17.12119	1.584520	0.1313712

6.6. Estimated Marginal Means (EMMs): Timepoint by Response

Timepoint	Response	EMM	SE	df	CI_low	CI_high
Baseline	Non-responder	7.768034	0.7469768	1.181674	1.116675	14.419394
Week 4	Non-responder	7.630888	0.7557088	1.241411	1.488961	13.772816
Baseline	Responder	6.765507	0.9531742	2.526365	3.383545	10.147469
Week 4	Responder	6.225066	1.1027300	3.834595	3.110603	9.339529

6.6.1. Pairwise Contrasts (Tukey): Timepoint by Response

contrast	Response	estimate	SE	df	t_ratio	p_value
Baseline - T4	Non-responder	0.1371461	0.2719992	96.05954	0.5042153	0.6152647
Baseline - T4	Responder	0.5404407	0.7915682	102.42181	0.6827469	0.4963081

6.7. Estimated Marginal Means (EMMs): Response by ECT

Response	ECT	EMM	SE	df	CI_low	CI_high
Non-responder	No ECT	8.077827	0.7294905	1.076940	0.2360986	15.919556
Responder	No ECT	7.749348	0.9849945	2.620218	4.3412650	11.157430
Non-responder	ECT	7.321095	0.8058136	1.551668	2.6925525	11.949638
Responder	ECT	5.241226	1.0522250	3.394564	2.1023552	8.380096

6.7.1. Pairwise Contrasts (Tukey): Response by ECT

contrast	ECT	estimate	SE	df	t_ratio	p_value
NO - YES	No ECT	0.3284798	0.7188862	19.44209	0.4569287	0.65278644
NO - YES	ECT	2.0798695	0.8596512	20.17583	2.4194341	0.02510499

6.8. Estimated Marginal Means (EMMs): ECT by Response

ECT	Response	EMM	SE	df	CI_low	CI_high
No ECT	Non-responder	8.077827	0.7294905	1.076940	0.2360986	15.919556
ECT	Non-responder	7.321095	0.8058136	1.551668	2.6925525	11.949638
No ECT	Responder	7.749348	0.9849945	2.620218	4.3412650	11.157430
ECT	Responder	5.241226	1.0522250	3.394564	2.1023552	8.380096

6.8.1. Pairwise Contrasts (Tukey): ECT by Response

contrast	Response	estimate	SE	df	t_ratio	p_value
NO - YES	Non-responder	0.7567321	0.4228795	86.66458	1.789474	0.0770316864
NO - YES	Responder	2.5081218	0.7295579	116.84366	3.437865	0.0008131574

7. Supplementary Results: Eosinophils (%)

7.1. Type III ANOVA (Satterthwaite)

Term	Sum Sq	Mean Sq	Num DF	Den DF	F value	p_value
Response	0.9743521036	0.9743521036	1	120.22874	1.9241230604	0.1679690
Cohort	0.0053936174	0.0053936174	1	117.79782	0.0106511635	0.9179762
Timepoint	0.0225248180	0.0225248180	1	88.07690	0.0444813755	0.8334478
ECT	0.0831417962	0.0831417962	1	113.60262	0.1641860747	0.6860934
Response:Cohort	0.4326724012	0.4326724012	1	116.62945	0.8544292578	0.3572109
Response:Timepoint	0.1493697459	0.1493697459	1	88.08115	0.2949711625	0.5884242
Cohort:Timepoint	0.1918358969	0.1918358969	1	87.85264	0.3788321199	0.5398202
Response:ECT	0.1804797195	0.1804797195	1	109.53739	0.3564062611	0.5517409
Timepoint:ECT	0.0003527169	0.0003527169	1	87.83380	0.0006965354	0.9790046
Response:Cohort:Timepoint	0.0476524789	0.0476524789	1	87.85715	0.0941027718	0.7597506
Response:Timepoint:ECT	1.2163837988	1.2163837988	1	87.84733	2.4020804276	0.1247693

7.2. Main effects (overall): Estimated Marginal Means (EMMs)

7.2.1. EMMs: Cohort (overall)

Cohort	EMM	SE	df	CI_low	CI_high
TRD	2.713802	0.2689329	2.112649	1.613837	3.813766
UDC	2.796470	0.8317367	69.697731	1.137497	4.455443

7.2.2. Pairwise Contrasts (Tukey): Cohort (overall)

contrast	estimate	SE	df	t_ratio	p_value
TRD - UDK	-0.08266873	0.9066007	115.6756	-0.09118539	0.927503

7.2.3. EMMs: ECT (overall)

ECT	EMM	SE	df	CI_low	CI_high
No ECT	2.655199	0.4497252	13.02791	1.683839	3.626560
ECT	2.855073	0.5322449	26.60784	1.762243	3.947903

7.2.4. Pairwise Contrasts (Tukey): ECT (overall)

contrast	estimate	SE	df	t_ratio	p_value
NO - YES	-0.1998737	0.5145657	112.9963	-0.3884319	0.6984273

7.2.5. EMMs: Response (overall)

Response	EMM	SE	df	CI_low	CI_high
Non-responder	3.274458	0.2890605	3.194466	2.3854219	4.163494
Responder	2.235814	0.7935840	65.317230	0.6510634	3.820565

7.2.6. Pairwise Contrasts (Tukey): Response (overall)

contrast	estimate	SE	df	t_ratio	p_value
NO - YES	1.038644	0.8487439	109.115	1.223742	0.2236854

7.2.7. EMMs: Timepoint (overall)

Timepoint	EMM	SE	df	CI_low	CI_high
Baseline	2.783904	0.4179612	10.64236	1.860191	3.707616
Week 4	2.726368	0.4649572	15.98539	1.740630	3.712107

7.2.8. Pairwise Contrasts (Tukey): Timepoint (overall)

contrast	estimate	SE	df	t_ratio	p_value
Baseline - T4	0.05753519	0.2746937	87.86865	0.2094521	0.8345801

7.3. Estimated Marginal Means (EMMs): Cohort by Timepoint

Cohort	Timepoint	EMM	SE	df	CI_low	CI_high
TRD	Baseline	2.830044	0.2784096	2.416763	1.809868	3.850219
UDC	Baseline	2.737764	0.8296497	72.148420	1.083945	4.391582
TRD	Week 4	2.597560	0.2847523	2.772795	1.647860	3.547260
UDC	Week 4	2.855177	0.9156472	100.481494	1.038666	4.671689

7.3.1. Pairwise Contrasts (Tukey): Cohort by Timepoint

contrast	Timepoint	estimate	SE	df	t_ratio	p_value
TRD - UDK	Baseline	0.09227993	0.9126307	123.1444	0.1011142	0.9196242
TRD - UDK	Week 4	-0.25761739	0.9870393	156.3383	-0.2610001	0.7944359

7.4. Estimated Marginal Means (EMMs): ECT by Timepoint

ECT	Timepoint	EMM	SE	df	CI_low	CI_high
No ECT	Baseline	2.681775	0.4506900	14.02106	1.715277	3.648272

ECT	Timepoint	EMM	SE	df	CI_low	CI_high
ECT	Baseline	2.886033	0.5364829	28.19478	1.787439	3.984626
No ECT	Week 4	2.628624	0.4922683	18.82797	1.597657	3.659591
ECT	Week 4	2.824113	0.5839588	38.92404	1.642871	4.005355

7.4.1. Pairwise Contrasts (Tukey): ECT by Timepoint

contrast	Timepoint	estimate	SE	df	t_ratio	p_value
NO - YES	Baseline	-0.2042580	0.5320757	129.3466	-0.3838890	0.7016910
NO - YES	Week 4	-0.1954894	0.5494826	142.7361	-0.3557699	0.7225381

7.5. Estimated Marginal Means (EMMs): Response by Timepoint

Response	Timepoint	EMM	SE	df	CI_low	CI_high
Non-responder	Baseline	3.377305	0.2972055	3.580810	2.5126010	4.242009
Responder	Baseline	2.190502	0.7851715	66.536933	0.6230927	3.757911
Non-responder	Week 4	3.171611	0.3073703	4.163085	2.3312383	4.011983
Responder	Week 4	2.281126	0.8821411	96.349962	0.5301711	4.032081

7.5.1. Pairwise Contrasts (Tukey): Response by Timepoint

contrast	Timepoint	estimate	SE	df	t_ratio	p_value
NO - YES	Baseline	1.1868031	0.8431397	113.9439	1.407600	0.1619719
NO - YES	Week 4	0.8904846	0.9383805	149.9612	0.948959	0.3441683

7.6. Estimated Marginal Means (EMMs): Timepoint by Response

Timepoint	Response	EMM	SE	df	CI_low	CI_high
Baseline	Non-responder	3.377305	0.2972055	3.580810	2.5126010	4.242009
Week 4	Non-responder	3.171611	0.3073703	4.163085	2.3312383	4.011983
Baseline	Responder	2.190502	0.7851715	66.536933	0.6230927	3.757911
Week 4	Responder	2.281126	0.8821411	96.349962	0.5301711	4.032081

7.6.1. Pairwise Contrasts (Tukey): Timepoint by Response

contrast	Response	estimate	SE	df	t_ratio	p_value
Baseline - T4	Non-responder	0.20569445	0.1771758	86.48476	1.1609624	0.2488517
Baseline - T4	Responder	-0.09062406	0.5198387	88.03867	-0.1743311	0.8620055

7.7. Estimated Marginal Means (EMMs): Response by ECT

Response	ECT	EMM	SE	df	CI_low	CI_high
Non-responder	No ECT	3.027791	0.2507002	1.813831	1.8357530	4.219830
Responder	No ECT	2.282607	0.8554609	77.894203	0.5794789	3.985735
Non-responder	ECT	3.521125	0.4946322	20.681923	2.4915166	4.550733
Responder	ECT	2.189021	0.9549854	89.935237	0.2917574	4.086285

7.7.1. Pairwise Contrasts (Tukey): Response by ECT

contrast	ECT	estimate	SE	df	t_ratio	p_value
NO - YES	No ECT	0.7451842	0.8833554	116.5609	0.8435836	0.4006300
NO - YES	ECT	1.3321035	1.0863599	102.1502	1.2262083	0.2229395

7.8. Estimated Marginal Means (EMMs): ECT by Response

ECT	Response	EMM	SE	df	CI_low	CI_high
No ECT	Non-responder	3.027791	0.2507002	1.813831	1.8357530	4.219830
ECT	Non-responder	3.521125	0.4946322	20.681923	2.4915166	4.550733
No ECT	Responder	2.282607	0.8554609	77.894203	0.5794789	3.985735
ECT	Responder	2.189021	0.9549854	89.935237	0.2917574	4.086285

7.8.1. Pairwise Contrasts (Tukey): ECT by Response

contrast	Response	estimate	SE	df	t_ratio	p_value
NO - YES	Non-responder	-0.49333339	0.5299049	98.44621	-0.9309848	0.3541389
NO - YES	Responder	0.09358598	0.8766520	112.98000	0.1067538	0.9151736

8. Supplementary Results: Basophils (%)

8.1. Type III ANOVA (Satterthwaite)

Term	Sum Sq	Mean Sq	Num DF	Den DF	F value	p_value
Response	0.0006827455	0.0006827455	1	104.82006	0.02805406	0.8673045
Cohort	0.0004393517	0.0004393517	1	104.50146	0.01805299	0.8933752
Timepoint	0.0047448152	0.0047448152	1	83.20728	0.19496480	0.6599615
ECT	0.0617280646	0.0617280646	1	113.86103	2.53641061	0.1140199
Response:Cohort	0.0018247885	0.0018247885	1	104.24204	0.07498069	0.7847602
Response:Timepoint	0.0117998812	0.0117998812	1	83.21262	0.48485797	0.4881705
Cohort:Timepoint	0.0007156273	0.0007156273	1	83.41451	0.02940518	0.8642622
Response:ECT	0.0318229929	0.0318229929	1	111.69781	1.30760906	0.2552729
Timepoint:ECT	0.0029391526	0.0029391526	1	90.31284	0.12076999	0.7290095
Response:Cohort:Timepoint	0.0046043150	0.0046043150	1	83.41459	0.18919163	0.6647135
Response:Timepoint:ECT	0.0072788641	0.0072788641	1	90.33169	0.29908905	0.5858026

8.2. Main effects (overall): Estimated Marginal Means (EMMs)

8.2.1. EMMs: Cohort (overall)

Cohort	EMM	SE	df	CI_low	CI_high
TRD	0.6998938	0.04645468	2.092431	0.5082427	0.8915449
UDC	0.7233577	0.20719140	85.951665	0.3114716	1.1352439

8.2.2. Pairwise Contrasts (Tukey): Cohort (overall)

contrast	estimate	SE	df	t_ratio	p_value
TRD - UDK	-0.02346392	0.2157364	104.1449	-0.108762	0.9136006

8.2.3. EMMs: ECT (overall)

ECT	EMM	SE	df	CI_low	CI_high
No ECT	0.7796722	0.1089037	25.58579	0.5556410	1.003703
ECT	0.6435793	0.1179995	40.08749	0.4051096	0.882049

8.2.4. Pairwise Contrasts (Tukey): ECT (overall)

contrast	estimate	SE	df	t_ratio	p_value
NO - YES	0.1360929	0.08909194	113.4523	1.527556	0.129406

8.2.5. EMMs: Response (overall)

Response	EMM	SE	df	CI_low	CI_high
Non-responder	0.7256557	0.0497981	3.143189	0.5711829	0.8801284
Responder	0.6975958	0.2036286	84.206967	0.2926727	1.1025190

8.2.6. Pairwise Contrasts (Tukey): Response (overall)

contrast	estimate	SE	df	t_ratio	p_value
NO - YES	0.02805986	0.2103769	94.3956	0.133379	0.8941776

8.2.7. EMMs: Timepoint (overall)

Timepoint	EMM	SE	df	CI_low	CI_high
Baseline	0.7250046	0.1082168	29.46406	0.5038276	0.9461816
Week 4	0.6982469	0.1092905	30.55178	0.4752148	0.9212791

8.2.8. Pairwise Contrasts (Tukey): Timepoint (overall)

contrast	estimate	SE	df	t_ratio	p_value
Baseline - T4	0.02675768	0.06066455	83.58085	0.441076	0.6602968

8.3. Estimated Marginal Means (EMMs): Cohort by Timepoint

Cohort	Timepoint	EMM	SE	df	CI_low	CI_high
TRD	Baseline	0.7078666	0.04906316	2.584520	0.5365190	0.8792143
UDC	Baseline	0.7421426	0.21529719	107.458130	0.3153618	1.1689233
TRD	Week 4	0.6919210	0.05069460	3.156497	0.5350201	0.8488218
UDC	Week 4	0.7045729	0.21565221	108.464367	0.2771335	1.1320123

8.3.1. Pairwise Contrasts (Tukey): Cohort by Timepoint

contrast	Timepoint	estimate	SE	df	t_ratio	p_value
TRD - UDK	Baseline	-0.03427593	0.2251146	130.8632	-0.15225991	0.8792164
TRD - UDK	Week 4	-0.01265191	0.2244414	130.9185	-0.05637065	0.9551325

8.4. Estimated Marginal Means (EMMs): ECT by Timepoint

ECT	Timepoint	EMM	SE	df	CI_low	CI_high
No ECT	Baseline	0.7993189	0.1131656	32.80581	0.5690299	1.0296079

ECT	Timepoint	EMM	SE	df	CI_low	CI_high
ECT	Baseline	0.6506903	0.1225733	49.25492	0.4044023	0.8969783
No ECT	Week 4	0.7600256	0.1136001	32.21696	0.5286909	0.9913603
ECT	Week 4	0.6364683	0.1257925	55.01585	0.3843761	0.8885604

8.4.1. Pairwise Contrasts (Tukey): ECT by Timepoint

contrast	Timepoint	estimate	SE	df	t_ratio	p_value
NO - YES	Baseline	0.1486286	0.09390319	138.5027	1.582785	0.1157513
NO - YES	Week 4	0.1235573	0.09838584	155.6311	1.255845	0.2110544

8.5. Estimated Marginal Means (EMMs): Response by Timepoint

Response	Timepoint	EMM	SE	df	CI_low	CI_high
Non-responder	Baseline	0.7179366	0.05208916	3.772409	0.5698070	0.8660662
Responder	Baseline	0.7320726	0.21079341	104.729859	0.3140956	1.1500496
Non-responder	Week 4	0.7333748	0.05469204	4.698799	0.5900301	0.8767195
Responder	Week 4	0.6631191	0.21238990	106.926229	0.2420776	1.0841606

8.5.1. Pairwise Contrasts (Tukey): Response by Timepoint

contrast	Timepoint	estimate	SE	df	t_ratio	p_value
NO - YES	Baseline	-0.01413599	0.2178319	117.6579	-0.06489404	0.9483685
NO - YES	Week 4	0.07025571	0.2200538	119.9742	0.31926601	0.7500800

8.6. Estimated Marginal Means (EMMs): Timepoint by Response

Timepoint	Response	EMM	SE	df	CI_low	CI_high
Baseline	Non-responder	0.7179366	0.05208916	3.772409	0.5698070	0.8660662
Week 4	Non-responder	0.7333748	0.05469204	4.698799	0.5900301	0.8767195
Baseline	Responder	0.7320726	0.21079341	104.729859	0.3140956	1.1500496
Week 4	Responder	0.6631191	0.21238990	106.926229	0.2420776	1.0841606

8.6.1. Pairwise Contrasts (Tukey): Timepoint by Response

contrast	Response	estimate	SE	df	t_ratio	p_value
Baseline - T4	Non-responder	-0.01543817	0.0385953	88.91338	-0.4000014	0.6901149
Baseline - T4	Responder	0.06895353	0.1150144	83.00906	0.5995210	0.5504584

8.7. Estimated Marginal Means (EMMs): Response by ECT

Response	ECT	EMM	SE	df	CI_low	CI_high
Non-responder	No ECT	0.7450012	0.04298194	1.735746	0.5302816	0.9597208
Responder	No ECT	0.8143433	0.21225455	86.293749	0.3924157	1.2362709
Non-responder	ECT	0.7063102	0.08549500	20.709158	0.5283615	0.8842590
Responder	ECT	0.5808484	0.22234840	96.627751	0.1395269	1.0221699

8.7.1. Pairwise Contrasts (Tukey): Response by ECT

contrast	ECT	estimate	SE	df	t_ratio	p_value
NO - YES	No ECT	-0.06934213	0.2153110	103.87422	-0.3220557	0.7480578
NO - YES	ECT	0.12546185	0.2404181	87.22227	0.5218486	0.6030984

8.8. Estimated Marginal Means (EMMs): ECT by Response

ECT	Response	EMM	SE	df	CI_low	CI_high
No ECT	Non-responder	0.7450012	0.04298194	1.735746	0.5302816	0.9597208
ECT	Non-responder	0.7063102	0.08549500	20.709158	0.5283615	0.8842590
No ECT	Responder	0.8143433	0.21225455	86.293749	0.3924157	1.2362709
ECT	Responder	0.5808484	0.22234840	96.627751	0.1395269	1.0221699

8.8.1. Pairwise Contrasts (Tukey): ECT by Response

contrast	Response	estimate	SE	df	t_ratio	p_value
NO - YES	Non-responder	0.03869096	0.09162031	98.90633	0.4222968	0.6737250
NO - YES	Responder	0.23349494	0.15206332	113.55927	1.5355113	0.1274398

9. Supplementary Results: NLR

9.1. Type III ANOVA (Satterthwaite)

Term	Sum Sq	Mean Sq	Num DF	Den DF	F value	p_value
Response	0.146415627	0.146415627	1	108.17345	0.34370563	0.55891939
Cohort	0.031520303	0.031520303	1	104.14485	0.07399282	0.78614808
Timepoint	0.131945972	0.131945972	1	74.07929	0.30973861	0.57951804
ECT	2.886497880	2.886497880	1	97.37863	6.77595412	0.01068335
Response:Cohort	0.013895259	0.013895259	1	103.23119	0.03261864	0.85703069
Response:Timepoint	1.663721095	1.663721095	1	74.07546	3.90552783	0.05185132
Cohort:Timepoint	0.016191310	0.016191310	1	73.43052	0.03800854	0.84596441
Response:ECT	0.008675849	0.008675849	1	95.75713	0.02036626	0.88681877
Timepoint:ECT	0.822505928	0.822505928	1	73.29492	1.93080427	0.16887716
Response:Cohort:Timepoint	0.237501333	0.237501333	1	73.43964	0.55752618	0.45764248
Response:Timepoint:ECT	1.643463643	1.643463643	1	73.29330	3.85797416	0.05330238

9.2. Main effects (overall): Estimated Marginal Means (EMMs)

9.2.1. EMMs: Cohort (overall)

Cohort	EMM	SE	df	CI_low	CI_high
TRD	2.265408	0.1717434	2.223791	1.593337	2.937478
UDC	2.126117	0.5395700	74.008045	1.051002	3.201231

9.2.2. Pairwise Contrasts (Tukey): Cohort (overall)

contrast	estimate	SE	df	t_ratio	p_value
TRD - UDK	0.139291	0.5848754	120.3803	0.2381551	0.8121657

9.2.3. EMMs: ECT (overall)

ECT	EMM	SE	df	CI_low	CI_high
No ECT	1.788222	0.2911980	13.82553	1.162924	2.413520
ECT	2.603302	0.3434878	28.45479	1.900205	3.306399

9.2.4. Pairwise Contrasts (Tukey): ECT (overall)

contrast	estimate	SE	df	t_ratio	p_value
NO - YES	-0.8150798	0.3261538	115.373	-2.499066	0.01385935

9.2.5. EMMs: Response (overall)

Response	EMM	SE	df	CI_low	CI_high
Non-responder	2.336839	0.1833181	3.262579	1.779122	2.894555
Responder	2.054686	0.5182120	69.961317	1.021134	3.088237

9.2.6. Pairwise Contrasts (Tukey): Response (overall)

contrast	estimate	SE	df	t_ratio	p_value
NO - YES	0.2821532	0.5523717	113.4491	0.5108032	0.6104815

9.2.7. EMMs: Timepoint (overall)

Timepoint	EMM	SE	df	CI_low	CI_high
Baseline	2.127152	0.2710710	12.01649	1.536629	2.717675
Week 4	2.264372	0.3276834	23.65518	1.587545	2.941199

9.2.8. Pairwise Contrasts (Tukey): Timepoint (overall)

contrast	estimate	SE	df	t_ratio	p_value
Baseline - T4	-0.1372199	0.2500558	93.06629	-0.5487569	0.5844864

9.3. Estimated Marginal Means (EMMs): Cohort by Timepoint

Cohort	Timepoint	EMM	SE	df	CI_low	CI_high
TRD	Baseline	2.171822	0.1832058	2.881405	1.574966	2.768678
UDC	Baseline	2.082482	0.5379336	80.626311	1.012088	3.152877
TRD	Week 4	2.358993	0.1925697	3.741677	1.809457	2.908529
UDC	Week 4	2.169751	0.6409312	126.351138	0.901401	3.438101

9.3.1. Pairwise Contrasts (Tukey): Cohort by Timepoint

contrast	Timepoint	estimate	SE	df	t_ratio	p_value
TRD - UDK	Baseline	0.08933987	0.5932587	137.4813	0.1505918	0.8805185
TRD - UDK	Week 4	0.18924221	0.6828222	179.6071	0.2771471	0.7819859

9.4. Estimated Marginal Means (EMMs): ECT by Timepoint

ECT	Timepoint	EMM	SE	df	CI_low	CI_high
No ECT	Baseline	1.824640	0.2929205	16.18318	1.204247	2.445033

ECT	Timepoint	EMM	SE	df	CI_low	CI_high
ECT	Baseline	2.429665	0.3490754	32.03703	1.718653	3.140676
No ECT	Week 4	1.751805	0.3427647	26.18944	1.047490	2.456120
ECT	Week 4	2.776939	0.4074499	54.31294	1.960159	3.593720

9.4.1. Pairwise Contrasts (Tukey): ECT by Timepoint

contrast	Timepoint	estimate	SE	df	t_ratio	p_value
NO - YES	Baseline	-0.6050249	0.3484167	146.6085	-1.736498	0.084576393
NO - YES	Week 4	-1.0251348	0.3708105	166.2601	-2.764579	0.006343374

9.5. Estimated Marginal Means (EMMs): Response by Timepoint

Response	Timepoint	EMM	SE	df	CI_low	CI_high
Non-responder	Baseline	2.024597	0.1939950	4.125253	1.4923691	2.556825
Responder	Baseline	2.229707	0.5084961	74.040810	1.2165161	3.242899
Non-responder	Week 4	2.649080	0.2061041	5.364036	2.1299073	3.168254
Responder	Week 4	1.879664	0.6249085	122.055766	0.6426006	3.116727

9.5.1. Pairwise Contrasts (Tukey): Response by Timepoint

contrast	Timepoint	estimate	SE	df	t_ratio	p_value
NO - YES	Baseline	-0.2051104	0.5463394	126.5933	-0.3754268	0.7079714
NO - YES	Week 4	0.7694169	0.6606612	169.1770	1.1646163	0.2458139

9.6. Estimated Marginal Means (EMMs): Timepoint by Response

Timepoint	Response	EMM	SE	df	CI_low	CI_high
Baseline	Non-responder	2.024597	0.1939950	4.125253	1.4923691	2.556825
Week 4	Non-responder	2.649080	0.2061041	5.364036	2.1299073	3.168254
Baseline	Responder	2.229707	0.5084961	74.040810	1.2165161	3.242899
Week 4	Responder	1.879664	0.6249085	122.055766	0.6426006	3.116727

9.6.1. Pairwise Contrasts (Tukey): Timepoint by Response

contrast	Response	estimate	SE	df	t_ratio	p_value
Baseline - T4	Non-responder	-0.6244835	0.1606357	89.70898	-3.8875763	0.0001937632
Baseline - T4	Responder	0.3500438	0.4732684	93.48399	0.7396306	0.4613774819

9.7. Estimated Marginal Means (EMMs): Response by ECT

Response	ECT	EMM	SE	df	CI_low	CI_high
Non-responder	No ECT	1.951543	0.1578620	1.817676	1.2026639	2.700423
Responder	No ECT	1.624901	0.5548082	81.846478	0.5211798	2.728622
Non-responder	ECT	2.722134	0.3135095	20.881556	2.0699300	3.374338
Responder	ECT	2.484470	0.6203346	95.326338	1.2530045	3.715936

9.7.1. Pairwise Contrasts (Tukey): Response by ECT

contrast	ECT	estimate	SE	df	t_ratio	p_value
NO - YES	No ECT	0.3266425	0.5712092	121.4355	0.5718438	0.5684848
NO - YES	ECT	0.2376640	0.7030442	104.7260	0.3380498	0.7360024

9.8. Estimated Marginal Means (EMMs): ECT by Response

ECT	Response	EMM	SE	df	CI_low	CI_high
No ECT	Non-responder	1.951543	0.1578620	1.817676	1.2026639	2.700423
ECT	Non-responder	2.722134	0.3135095	20.881556	2.0699300	3.374338
No ECT	Responder	1.624901	0.5548082	81.846478	0.5211798	2.728622
ECT	Responder	2.484470	0.6203346	95.326338	1.2530045	3.715936

9.8.1. Pairwise Contrasts (Tukey): ECT by Response

contrast	Response	estimate	SE	df	t_ratio	p_value
NO - YES	Non-responder	-0.7705906	0.3346568	98.76981	-2.30263	0.02339785
NO - YES	Responder	-0.8595691	0.5577451	116.09028	-1.54115	0.12600165

10. Supplementary Results: MLR

10.1. Type III ANOVA (Satterthwaite)

Term	Sum Sq	Mean Sq	Num DF	Den DF	F value	p_value
Response	0.0108983515	0.0108983515	1	123.34213	2.8128428	0.09604507
Cohort	0.0206575289	0.0206575289	1	119.60526	5.3316671	0.02265758
Timepoint	0.0010679672	0.0010679672	1	92.75470	0.2756402	0.60082580
ECT	0.0006685561	0.0006685561	1	106.68836	0.1725530	0.67868703
Response:Cohort	0.0134966475	0.0134966475	1	116.25041	3.4834579	0.06450503
Response:Timepoint	0.0184508620	0.0184508620	1	92.73647	4.7621308	0.03161920
Cohort:Timepoint	0.0005621215	0.0005621215	1	91.56655	0.1450824	0.70416204
Response:ECT	0.0035588125	0.0035588125	1	104.80804	0.9185224	0.34006887
Timepoint:ECT	0.0005688409	0.0005688409	1	90.54301	0.1468167	0.70249431
Response:Cohort:Timepoint	0.0014172222	0.0014172222	1	91.59872	0.3657822	0.54680766
Response:Timepoint:ECT	0.0041297503	0.0041297503	1	90.58627	1.0658803	0.30462635

10.2. Main effects (overall): Estimated Marginal Means (EMMs)

10.2.1. EMMs: Cohort (overall)

Cohort	EMM	SE	df	CI_low	CI_high
TRD	0.2916461	0.03279893	1.203995	0.009730807	0.5735614
UDC	0.2013960	0.05074049	5.136693	0.072000570	0.3307915

10.2.2. Pairwise Contrasts (Tukey): Cohort (overall)

contrast	estimate	SE	df	t_ratio	p_value
TRD - UDK	0.09025008	0.04357546	122.5177	2.071122	0.04044688

10.2.3. EMMs: ECT (overall)

ECT	EMM	SE	df	CI_low	CI_high
No ECT	0.2513593	0.03748021	1.801741	0.07183721	0.4308814
ECT	0.2416829	0.03989427	2.332147	0.09145609	0.3919096

10.2.4. Pairwise Contrasts (Tukey): ECT (overall)

contrast	estimate	SE	df	t_ratio	p_value
NO - YES	0.009676449	0.02430482	113.5022	0.3981288	0.6912831

10.2.5. EMMs: Response (overall)

Response	EMM	SE	df	CI_low	CI_high
Non-responder	0.2775520	0.03314227	1.262710	0.01619742	0.5389066
Responder	0.2154901	0.04971064	4.671256	0.08495202	0.3460283

10.2.6. Pairwise Contrasts (Tukey): Response (overall)

contrast	estimate	SE	df	t_ratio	p_value
NO - YES	0.06206187	0.04167976	114.5921	1.489017	0.1392299

10.2.7. EMMs: Timepoint (overall)

Timepoint	EMM	SE	df	CI_low	CI_high
Baseline	0.2525522	0.03664802	1.729438	0.06870071	0.4364038
Week 4	0.2404899	0.04040451	2.394626	0.09140740	0.3895724

10.2.8. Pairwise Contrasts (Tukey): Timepoint (overall)

contrast	estimate	SE	df	t_ratio	p_value
Baseline - T4	0.01206233	0.02343693	100.8589	0.5146719	0.6079083

10.3. Estimated Marginal Means (EMMs): Cohort by Timepoint

Cohort	Timepoint	EMM	SE	df	CI_low	CI_high
TRD	Baseline	0.3022461	0.03331996	1.283130	0.04670615	0.5577860
UDC	Baseline	0.2028584	0.05065257	5.570097	0.07655986	0.3291569
TRD	Week 4	0.2810461	0.03379329	1.353796	0.04393448	0.5181578
UDC	Week 4	0.1999337	0.06020600	10.107982	0.06598037	0.3338870

10.3.1. Pairwise Contrasts (Tukey): Cohort by Timepoint

contrast	Timepoint	estimate	SE	df	t_ratio	p_value
TRD - UDK	Baseline	0.09938770	0.04449154	151.4675	2.233856	0.02695738
TRD - UDK	Week 4	0.08111245	0.05480328	183.3527	1.480066	0.14057196

10.4. Estimated Marginal Means (EMMs): ECT by Timepoint

ECT	Timepoint	EMM	SE	df	CI_low	CI_high
No ECT	Baseline	0.2600759	0.03765089	1.930806	0.09238461	0.4277672

ECT	Timepoint	EMM	SE	df	CI_low	CI_high
ECT	Baseline	0.2450286	0.04041865	2.530089	0.10176784	0.3882894
No ECT	Week 4	0.2426427	0.04111895	2.528705	0.09684357	0.3884419
ECT	Week 4	0.2383371	0.04471088	3.650280	0.10936574	0.3673085

10.4.1. Pairwise Contrasts (Tukey): ECT by Timepoint

contrast	Timepoint	estimate	SE	df	t_ratio	p_value
NO - YES	Baseline	0.015047292	0.02702231	161.1268	0.556847	0.5784046
NO - YES	Week 4	0.004305607	0.02914727	174.0143	0.147719	0.8827355

10.5. Estimated Marginal Means (EMMs): Response by Timepoint

Response	Timepoint	EMM	SE	df	CI_low	CI_high
Non-responder	Baseline	0.2585135	0.03369495	1.346411	0.02000344	0.4970235
Responder	Baseline	0.2465910	0.04901071	4.847474	0.11940329	0.3737788
Non-responder	Week 4	0.2965906	0.03428954	1.447886	0.07955435	0.5136268
Responder	Week 4	0.1843893	0.05935430	9.403010	0.05099262	0.3177859

10.5.1. Pairwise Contrasts (Tukey): Response by Timepoint

contrast	Timepoint	estimate	SE	df	t_ratio	p_value
NO - YES	Baseline	0.01192242	0.04126123	139.1585	0.2889496	0.77304991
NO - YES	Week 4	0.11220132	0.05354730	170.4428	2.0953684	0.03761603

10.6. Estimated Marginal Means (EMMs): Timepoint by Response

Timepoint	Response	EMM	SE	df	CI_low	CI_high
Baseline	Non-responder	0.2585135	0.03369495	1.346411	0.02000344	0.4970235
Week 4	Non-responder	0.2965906	0.03428954	1.447886	0.07955435	0.5136268
Baseline	Responder	0.2465910	0.04901071	4.847474	0.11940329	0.3737788
Week 4	Responder	0.1843893	0.05935430	9.403010	0.05099262	0.3177859

10.6.1. Pairwise Contrasts (Tukey): Timepoint by Response

contrast	Response	estimate	SE	df	t_ratio	p_value
Baseline - T4	Non-responder	-0.03807712	0.01511961	95.15639	-2.518393	0.01345801
Baseline - T4	Responder	0.06220178	0.04433254	101.57245	1.403073	0.16364466

10.7. Estimated Marginal Means (EMMs): Response by ECT

Response	ECT	EMM	SE	df	CI_low	CI_high
Non-responder	No ECT	0.2712859	0.03249542	1.163764	-0.02672025	0.5692920
Responder	No ECT	0.2314327	0.05187536	5.606717	0.10230845	0.3605570
Non-responder	ECT	0.2838182	0.03775260	2.065197	0.12619836	0.4414380
Responder	ECT	0.1995476	0.05586773	7.650532	0.06968478	0.3294103

10.7.1. Pairwise Contrasts (Tukey): Response by ECT

contrast	ECT	estimate	SE	df	t_ratio	p_value
NO - YES	No ECT	0.03985313	0.04330064	126.1427	0.9203822	0.3591299
NO - YES	ECT	0.08427061	0.05221793	105.6916	1.6138251	0.1095471

10.8. Estimated Marginal Means (EMMs): ECT by Response

ECT	Response	EMM	SE	df	CI_low	CI_high
No ECT	Non-responder	0.2712859	0.03249542	1.163764	-0.02672025	0.5692920
ECT	Non-responder	0.2838182	0.03775260	2.065197	0.12619836	0.4414380
No ECT	Responder	0.2314327	0.05187536	5.606717	0.10230845	0.3605570
ECT	Responder	0.1995476	0.05586773	7.650532	0.06968478	0.3294103

10.8.1. Pairwise Contrasts (Tukey): ECT by Response

contrast	Response	estimate	SE	df	t_ratio	p_value
NO - YES	Non-responder	-0.01253229	0.02384914	96.82408	-0.5254817	0.6004497
NO - YES	Responder	0.03188518	0.04171235	115.74809	0.7644064	0.4461806

11. Supplementary Results: NPR

11.1. Type III ANOVA (Satterthwaite)

Term	Sum Sq	Mean Sq	Num DF	Den DF	F value	p_value
Response	0.00000043139690	0.00000043139690	1	92.12809	0.075416996	0.7842219
Cohort	0.00000977961644	0.00000977961644	1	88.51920	1.709676861	0.1944132
Timepoint	0.00000058972954	0.00000058972954	1	67.54491	0.103096778	0.7491360
ECT	0.00000004013831	0.00000004013831	1	107.02521	0.007016998	0.9333978
Response:Cohort	0.00000079890954	0.00000079890954	1	88.59699	0.139665718	0.7095064
Response:Timepoint	0.00000010396325	0.00000010396325	1	67.76360	0.018174902	0.8931583
Cohort:Timepoint	0.00000073962024	0.00000073962024	1	64.81825	0.129300736	0.7203270
Response:ECT	0.00000008071155	0.00000008071155	1	107.54107	0.014110029	0.9056668
Timepoint:ECT	0.00000134263619	0.00000134263619	1	75.76195	0.234720251	0.6294434
Response:Cohort:Timepoint	0.00000047897985	0.00000047897985	1	64.81270	0.083735469	0.7732213
Response:Timepoint:ECT	0.00000058434804	0.00000058434804	1	76.00704	0.102155983	0.7501344

11.2. Main effects (overall): Estimated Marginal Means (EMMs)

11.2.1. EMMs: Cohort (overall)

Cohort	EMM	SE	df	CI_low	CI_high
TRD	0.01621807	0.002124175	1.249208	-0.000853926	0.03329006
UDC	0.01286415	0.003600300	6.117059	0.004095238	0.02163306

11.2.2. Pairwise Contrasts (Tukey): Cohort (overall)

contrast	estimate	SE	df	t_ratio	p_value
TRD - UDK	0.003353919	0.003103163	90.85657	1.080807	0.2826437

11.2.3. EMMs: ECT (overall)

ECT	EMM	SE	df	CI_low	CI_high
No ECT	0.01459863	0.002527164	1.992366	0.003685093	0.02551216
ECT	0.01448359	0.002697547	2.616996	0.005142332	0.02382484

11.2.4. Pairwise Contrasts (Tukey): ECT (overall)

contrast	estimate	SE	df	t_ratio	p_value
NO - YES	0.000115041	0.001416932	108.9811	0.08119021	0.9354396

11.2.5. EMMs: Response (overall)

Response	EMM	SE	df	CI_low	CI_high
Non-responder	0.01488817	0.002177153	1.373422	-0.00004292802	0.02981926
Responder	0.01419405	0.003550642	5.755643	0.00541574275	0.02297236

11.2.6. Pairwise Contrasts (Tukey): Response (overall)

contrast	estimate	SE	df	t_ratio	p_value
NO - YES	0.0006941172	0.003061889	72.74432	0.2266957	0.8212962

11.2.7. EMMs: Timepoint (overall)

Timepoint	EMM	SE	df	CI_low	CI_high
Baseline	0.01470420	0.002548099	2.069714	0.004087004	0.02532140
Week 4	0.01437802	0.002585783	2.207281	0.004195900	0.02456013

11.2.8. Pairwise Contrasts (Tukey): Timepoint (overall)

contrast	estimate	SE	df	t_ratio	p_value
Baseline - T4	0.0003261862	0.001019425	70.4295	0.3199706	0.7499379

11.3. Estimated Marginal Means (EMMs): Cohort by Timepoint

Cohort	Timepoint	EMM	SE	df	CI_low	CI_high
TRD	Baseline	0.01655499	0.002132674	1.264714	-0.0002162730	0.03332626
UDC	Baseline	0.01285341	0.003705544	7.090723	0.0041138692	0.02159296
TRD	Week 4	0.01588115	0.002172303	1.369628	0.0009182668	0.03084402
UDC	Week 4	0.01287489	0.003731715	7.341651	0.0041333475	0.02161642

11.3.1. Pairwise Contrasts (Tukey): Cohort by Timepoint

contrast	Timepoint	estimate	SE	df	t_ratio	p_value
TRD - UDK	Baseline	0.003701579	0.003253839	116.5590	1.1376034	0.2576192
TRD - UDK	Week 4	0.003006259	0.003247168	116.3753	0.9258095	0.3564609

11.4. Estimated Marginal Means (EMMs): ECT by Timepoint

ECT	Timepoint	EMM	SE	df	CI_low	CI_high
No ECT	Baseline	0.01459495	0.002571749	2.158383	0.004272432	0.02491747

ECT	Timepoint	EMM	SE	df	CI_low	CI_high
ECT	Baseline	0.01481345	0.002729331	2.745637	0.005654043	0.02397287
No ECT	Week 4	0.01460231	0.002574355	2.162877	0.004287917	0.02491670
ECT	Week 4	0.01415372	0.002856046	3.373532	0.005608156	0.02269929

11.4.1. Pairwise Contrasts (Tukey): ECT by Timepoint

contrast	Timepoint	estimate	SE	df	t_ratio	p_value
NO - YES	Baseline	-0.0002185044	0.001468007	121.9779	-0.1488442	0.8819223
NO - YES	Week 4	0.0004485865	0.001680331	157.2635	0.2669632	0.7898473

11.5. Estimated Marginal Means (EMMs): Response by Timepoint

Response	Timepoint	EMM	SE	df	CI_low	CI_high
Non-responder	Baseline	0.01498285	0.002185511	1.387923	0.0002382375	0.02972747
Responder	Baseline	0.01442555	0.003639343	6.546702	0.0056975949	0.02315351
Non-responder	Week 4	0.01479348	0.002254374	1.583448	0.0021868814	0.02740008
Responder	Week 4	0.01396255	0.003696639	7.057779	0.0052358718	0.02268923

11.5.1. Pairwise Contrasts (Tukey): Response by Timepoint

contrast	Timepoint	estimate	SE	df	t_ratio	p_value
NO - YES	Baseline	0.0005573005	0.003173532	81.97646	0.1756089	0.8610343
NO - YES	Week 4	0.0008309340	0.003278656	108.48318	0.2534374	0.8004099

11.6. Estimated Marginal Means (EMMs): Timepoint by Response

Timepoint	Response	EMM	SE	df	CI_low	CI_high
Baseline	Non-responder	0.01498285	0.002185511	1.387923	0.0002382375	0.02972747
Week 4	Non-responder	0.01479348	0.002254374	1.583448	0.0021868814	0.02740008
Baseline	Responder	0.01442555	0.003639343	6.546702	0.0056975949	0.02315351
Week 4	Responder	0.01396255	0.003696639	7.057779	0.0052358718	0.02268923

11.6.1. Pairwise Contrasts (Tukey): Timepoint by Response

contrast	Response	estimate	SE	df	t_ratio	p_value
Baseline - T4	Non-responder	0.0001893694	0.0008702507	82.67513	0.2176032	0.8282744
Baseline - T4	Responder	0.0004630029	0.0018416521	68.15381	0.2514063	0.8022565

11.7. Estimated Marginal Means (EMMs): Response by ECT

Response	ECT	EMM	SE	df	CI_low	CI_high
Non-responder	No ECT	0.01486394	0.002089012	1.175902	-0.003912816	0.03364069
Responder	No ECT	0.01433332	0.003630154	6.417070	0.005588738	0.02307790
Non-responder	ECT	0.01491240	0.002541809	2.457934	0.005714078	0.02411072
Responder	ECT	0.01405478	0.003834207	8.226258	0.005255232	0.02285433

11.7.1. Pairwise Contrasts (Tukey): Response by ECT

contrast	ECT	estimate	SE	df	t_ratio	p_value
NO - YES	No ECT	0.0005306154	0.003088324	90.36379	0.1718134	0.8639684
NO - YES	ECT	0.0008576191	0.003635498	68.64836	0.2359014	0.8142116

11.8. Estimated Marginal Means (EMMs): ECT by Response

ECT	Response	EMM	SE	df	CI_low	CI_high
No ECT	Non-responder	0.01486394	0.002089012	1.175902	-0.003912816	0.03364069
ECT	Non-responder	0.01491240	0.002541809	2.457934	0.005714078	0.02411072
No ECT	Responder	0.01433332	0.003630154	6.417070	0.005588738	0.02307790
ECT	Responder	0.01405478	0.003834207	8.226258	0.005255232	0.02285433

11.8.1. Pairwise Contrasts (Tukey): ECT by Response

contrast	Response	estimate	SE	df	t_ratio	p_value
NO - YES	Non-responder	-0.00004846086	0.001639985	105.4361	-0.02954957	0.9764821
NO - YES	Responder	0.00027854292	0.002308701	107.4970	0.12064917	0.9041941

12. Supplementary Results: Neutrophils (10^9/L)

12.1. Type III ANOVA (Satterthwaite)

Term	Sum Sq	Mean Sq	Num DF	Den DF	F value	p_value
Response	0.092028752	0.092028752	1	120.37700	0.13338087	0.71559298
Cohort	0.007126115	0.007126115	1	116.01824	0.01032816	0.91922775
Timepoint	0.129532662	0.129532662	1	90.40658	0.18773675	0.66583906
ECT	1.299506993	1.299506993	1	108.66879	1.88342624	0.17277129
Response:Cohort	0.494826763	0.494826763	1	115.61494	0.71717176	0.39882275
Response:Timepoint	1.981315161	1.981315161	1	90.49330	2.87159745	0.09359425
Cohort:Timepoint	0.272914297	0.272914297	1	89.66079	0.39554535	0.53099792
Response:ECT	0.081576454	0.081576454	1	106.03966	0.11823194	0.73164094
Timepoint:ECT	0.195526464	0.195526464	1	89.58551	0.28338414	0.59580952
Response:Cohort:Timepoint	0.511819126	0.511819126	1	89.70049	0.74179945	0.39138153
Response:Timepoint:ECT	0.596460267	0.596460267	1	89.53865	0.86447316	0.35498986

12.2. Main effects (overall): Estimated Marginal Means (EMMs)

12.2.1. EMMs: Cohort (overall)

Cohort	EMM	SE	df	CI_low	CI_high
TRD	4.199380	0.5194263	1.516562	1.121521	7.277238
UDC	4.259051	0.8001217	6.972076	2.365526	6.152575

12.2.2. Pairwise Contrasts (Tukey): Cohort (overall)

contrast	estimate	SE	df	t_ratio	p_value
TRD - UDK	-0.05967113	0.6755599	121.2368	-0.0883284	0.9297614

12.2.3. EMMs: ECT (overall)

ECT	EMM	SE	df	CI_low	CI_high
No ECT	3.982849	0.5990444	2.359792	1.747642	6.218056
ECT	4.475581	0.6257630	3.129434	2.529920	6.421243

12.2.4. Pairwise Contrasts (Tukey): ECT (overall)

contrast	estimate	SE	df	t_ratio	p_value
NO - YES	-0.4927324	0.3704742	114.7526	-1.330004	0.1861543

12.2.5. EMMs: Response (overall)

Response	EMM	SE	df	CI_low	CI_high
Non-responder	4.128299	0.5346222	1.617881	1.220223	7.036375
Responder	4.330131	0.7748313	6.278020	2.454355	6.205908

12.2.6. Pairwise Contrasts (Tukey): Response (overall)

contrast	estimate	SE	df	t_ratio	p_value
NO - YES	-0.2018327	0.6393383	112.5285	-0.3156899	0.7528226

12.2.7. EMMs: Timepoint (overall)

Timepoint	EMM	SE	df	CI_low	CI_high
Baseline	4.296731	0.5787971	2.227472	2.034884	6.558578
Week 4	4.161699	0.6299230	2.978947	2.148964	6.174434

12.2.8. Pairwise Contrasts (Tukey): Timepoint (overall)

contrast	estimate	SE	df	t_ratio	p_value
Baseline - T4	0.1350322	0.3162315	95.32475	0.4270043	0.6703391

12.3. Estimated Marginal Means (EMMs): Cohort by Timepoint

Cohort	Timepoint	EMM	SE	df	CI_low	CI_high
TRD	Baseline	4.165112	0.5226834	1.593678	1.266575	7.063648
UDC	Baseline	4.428351	0.7952892	7.465759	2.571314	6.285387
TRD	Week 4	4.233647	0.5339743	1.694107	1.488999	6.978296
UDC	Week 4	4.089750	0.9145777	12.417857	2.104468	6.075032

12.3.1. Pairwise Contrasts (Tukey): Cohort by Timepoint

contrast	Timepoint	estimate	SE	df	t_ratio	p_value
TRD - UDK	Baseline	-0.2632392	0.6865431	142.9697	-0.3834271	0.7019727
TRD - UDK	Week 4	0.1438970	0.8099073	183.4579	0.1776709	0.8591775

12.4. Estimated Marginal Means (EMMs): ECT by Timepoint

ECT	Timepoint	EMM	SE	df	CI_low	CI_high
No ECT	Baseline	4.101223	0.6010685	2.497479	1.951059	6.251388

ECT	Timepoint	EMM	SE	df	CI_low	CI_high
ECT	Baseline	4.492239	0.6245954	3.332872	2.612222	6.372255
No ECT	Week 4	3.864474	0.6404375	3.165085	1.885156	5.843792
ECT	Week 4	4.458924	0.6902494	4.649151	2.643540	6.274307

12.4.1. Pairwise Contrasts (Tukey): ECT by Timepoint

contrast	Timepoint	estimate	SE	df	t_ratio	p_value
NO - YES	Baseline	-0.3910154	0.4034617	151.7080	-0.9691511	0.3340123
NO - YES	Week 4	-0.5944494	0.4312737	170.5983	-1.3783577	0.1698978

12.5. Estimated Marginal Means (EMMs): Response by Timepoint

Response	Timepoint	EMM	SE	df	CI_low	CI_high
Non-responder	Baseline	3.931866	0.5341294	1.699804	1.197369	6.666363
Responder	Baseline	4.661597	0.7643788	6.464858	2.823351	6.499842
Non-responder	Week 4	4.324732	0.5544832	1.829950	1.713414	6.936050
Responder	Week 4	3.998666	0.8916670	11.546628	2.047396	5.949936

12.5.1. Pairwise Contrasts (Tukey): Response by Timepoint

contrast	Timepoint	estimate	SE	df	t_ratio	p_value
NO - YES	Baseline	-0.7297310	0.6317548	131.0585	-1.1550858	0.2501569
NO - YES	Week 4	0.3260657	0.7860227	168.3684	0.4148299	0.6787945

12.6. Estimated Marginal Means (EMMs): Timepoint by Response

Timepoint	Response	EMM	SE	df	CI_low	CI_high
Baseline	Non-responder	3.931866	0.5341294	1.699804	1.197369	6.666363
Week 4	Non-responder	4.324732	0.5544832	1.829950	1.713414	6.936050
Baseline	Responder	4.661597	0.7643788	6.464858	2.823351	6.499842
Week 4	Responder	3.998666	0.8916670	11.546628	2.047396	5.949936

12.6.1. Pairwise Contrasts (Tukey): Timepoint by Response

contrast	Response	estimate	SE	df	t_ratio	p_value
Baseline - T4	Non-responder	-0.3928661	0.2054458	92.37396	-1.912262	0.05894136
Baseline - T4	Responder	0.6629305	0.5976919	95.69560	1.109151	0.27014471

12.7. Estimated Marginal Means (EMMs): Response by ECT

Response	ECT	EMM	SE	df	CI_low	CI_high
Non-responder	No ECT	3.943197	0.5192452	1.470159	0.7287004	7.157694
Responder	No ECT	4.022501	0.8158944	7.699596	2.1281903	5.916811
Non-responder	ECT	4.313400	0.6120399	2.807763	2.2879562	6.338844
Responder	ECT	4.637762	0.8596034	10.553608	2.7359784	6.539546

12.7.1. Pairwise Contrasts (Tukey): Response by ECT

contrast	ECT	estimate	SE	df	t_ratio	p_value
NO - YES	No ECT	-0.07930331	0.6596834	122.5331	-0.1202142	0.9045104
NO - YES	ECT	-0.32436202	0.8128373	103.8575	-0.3990491	0.6906763

12.8. Estimated Marginal Means (EMMs): ECT by Response

ECT	Response	EMM	SE	df	CI_low	CI_high
No ECT	Non-responder	3.943197	0.5192452	1.470159	0.7287004	7.157694
ECT	Non-responder	4.313400	0.6120399	2.807763	2.2879562	6.338844
No ECT	Responder	4.022501	0.8158944	7.699596	2.1281903	5.916811
ECT	Responder	4.637762	0.8596034	10.553608	2.7359784	6.539546

12.8.1. Pairwise Contrasts (Tukey): ECT by Response

contrast	Response	estimate	SE	df	t_ratio	p_value
NO - YES	Non-responder	-0.3702031	0.3809634	98.04978	-0.9717550	0.3335636
NO - YES	Responder	-0.6152618	0.6385522	116.00153	-0.9635261	0.3372879

13. Supplementary Results: Monocytes (10^9/L)

13.1. Type III ANOVA (Satterthwaite)

Term	Sum Sq	Mean Sq	Num DF	Den DF	F value	p_value
Response	0.00562887956	0.00562887956	1	130.32407	0.417426242	0.51936017
Cohort	0.04362339956	0.04362339956	1	125.48525	3.235022449	0.07448309
Timepoint	0.01549735594	0.01549735594	1	102.03087	1.149252348	0.28623487
ECT	0.06314200309	0.06314200309	1	117.03086	4.682482327	0.03250383
Response:Cohort	0.02799234357	0.02799234357	1	124.24693	2.075855178	0.15216139
Response:Timepoint	0.02177970685	0.02177970685	1	102.10175	1.615138694	0.20665891
Cohort:Timepoint	0.00004040839	0.00004040839	1	101.15516	0.002996604	0.95645258
Response:ECT	0.01596683776	0.01596683776	1	117.30418	1.184068163	0.27876000
Timepoint:ECT	0.00025852265	0.00025852265	1	99.95209	0.019171513	0.89015448
Response:Cohort:Timepoint	0.00353794049	0.00353794049	1	101.18892	0.262366460	0.60961519
Response:Timepoint:ECT	0.00360735262	0.00360735262	1	99.84825	0.267513922	0.60614825

13.2. Main effects (overall): Estimated Marginal Means (EMMs)

13.2.1. EMMs: Cohort (overall)

Cohort	EMM	SE	df	CI_low	CI_high
TRD	0.5600048	0.07675572	2.004459	0.2304547	0.8895549
UDC	0.4269988	0.10669092	5.751400	0.1631756	0.6908219

13.2.2. Pairwise Contrasts (Tukey): Cohort (overall)

contrast	estimate	SE	df	t_ratio	p_value
TRD - UDK	0.133006	0.08322405	122.3567	1.598168	0.1125848

13.2.3. EMMs: ECT (overall)

ECT	EMM	SE	df	CI_low	CI_high
No ECT	0.5418034	0.08408770	2.631196	0.2516754	0.8319315
ECT	0.4452001	0.08826387	3.173435	0.1727932	0.7176071

13.2.4. Pairwise Contrasts (Tukey): ECT (overall)

contrast	estimate	SE	df	t_ratio	p_value
NO - YES	0.09660329	0.04582532	114.916	2.108076	0.03719761

13.2.5. EMMs: Response (overall)

Response	EMM	SE	df	CI_low	CI_high
Non-responder	0.5159950	0.07741597	2.072560	0.1938304	0.8381596
Responder	0.4710086	0.10469597	5.299186	0.2063857	0.7356315

13.2.6. Pairwise Contrasts (Tukey): Response (overall)

contrast	estimate	SE	df	t_ratio	p_value
NO - YES	0.04498637	0.07928691	105.784	0.5673871	0.5716534

13.2.7. EMMs: Timepoint (overall)

Timepoint	EMM	SE	df	CI_low	CI_high
Baseline	0.5165327	0.08296312	2.560727	0.2249400	0.8081255
Week 4	0.4704708	0.08877761	3.190842	0.1972639	0.7436777

13.2.8. Pairwise Contrasts (Tukey): Timepoint (overall)

contrast	estimate	SE	df	t_ratio	p_value
Baseline - T4	0.04606193	0.04365942	100.0412	1.055028	0.2939536

13.3. Estimated Marginal Means (EMMs): Cohort by Timepoint

Cohort	Timepoint	EMM	SE	df	CI_low	CI_high
TRD	Baseline	0.5842624	0.07757909	2.088666	0.2636845	0.9048403
UDC	Baseline	0.4488031	0.10654411	6.181292	0.1899413	0.7076648
TRD	Week 4	0.5357472	0.07819417	2.159952	0.2220880	0.8494064
UDC	Week 4	0.4051944	0.12261465	9.863819	0.1314798	0.6789090

13.3.1. Pairwise Contrasts (Tukey): Cohort by Timepoint

contrast	Timepoint	estimate	SE	df	t_ratio	p_value
TRD - UDK	Baseline	0.1354593	0.08490469	152.4410	1.595428	0.1126876
TRD - UDK	Week 4	0.1305528	0.10378586	185.9345	1.257905	0.2100035

13.4. Estimated Marginal Means (EMMs): ECT by Timepoint

ECT	Timepoint	EMM	SE	df	CI_low	CI_high
No ECT	Baseline	0.5666496	0.08444752	2.759551	0.2841542	0.8491450

ECT	Timepoint	EMM	SE	df	CI_low	CI_high
ECT	Baseline	0.4664159	0.08904406	3.373586	0.1999895	0.7328422
No ECT	Week 4	0.5169572	0.08968933	3.331574	0.2469425	0.7869719
ECT	Week 4	0.4239844	0.09602029	4.381341	0.1662958	0.6816731

13.4.1. Pairwise Contrasts (Tukey): ECT by Timepoint

contrast	Timepoint	estimate	SE	df	t_ratio	p_value
NO - YES	Baseline	0.10023378	0.05088163	160.0093	1.969941	0.05057127
NO - YES	Week 4	0.09297279	0.05479312	176.5231	1.696797	0.09149765

13.5. Estimated Marginal Means (EMMs): Response by Timepoint

Response	Timepoint	EMM	SE	df	CI_low	CI_high
Non-responder	Baseline	0.5117315	0.07824761	2.159899	0.1978512	0.8256117
Responder	Baseline	0.5213340	0.10357823	5.494794	0.2621300	0.7805381
Non-responder	Week 4	0.5202585	0.07913763	2.264514	0.2151700	0.8253470
Responder	Week 4	0.4206832	0.12084324	9.244726	0.1484159	0.6929504

13.5.1. Pairwise Contrasts (Tukey): Response by Timepoint

contrast	Timepoint	estimate	SE	df	t_ratio	p_value
NO - YES	Baseline	-0.009602595	0.07855418	132.7236	-0.1222417	0.9028925
NO - YES	Week 4	0.099575330	0.10102400	160.5306	0.9856601	0.3257832

13.6. Estimated Marginal Means (EMMs): Timepoint by Response

Timepoint	Response	EMM	SE	df	CI_low	CI_high
Baseline	Non-responder	0.5117315	0.07824761	2.159899	0.1978512	0.8256117
Week 4	Non-responder	0.5202585	0.07913763	2.264514	0.2151700	0.8253470
Baseline	Responder	0.5213340	0.10357823	5.494794	0.2621300	0.7805381
Week 4	Responder	0.4206832	0.12084324	9.244726	0.1484159	0.6929504

13.6.1. Pairwise Contrasts (Tukey): Timepoint by Response

contrast	Response	estimate	SE	df	t_ratio	p_value
Baseline - T4	Non-responder	-0.008527029	0.02824850	94.6852	-0.3018578	0.7634225
Baseline - T4	Responder	0.100650896	0.08257175	100.7490	1.2189508	0.2257092

13.7. Estimated Marginal Means (EMMs): Response by ECT

Response	ECT	EMM	SE	df	CI_low	CI_high
Non-responder	No ECT	0.5400835	0.07624500	1.954143	0.2045390	0.8756280
Responder	No ECT	0.5435234	0.10835200	6.188397	0.2803399	0.8067069
Non-responder	ECT	0.4919065	0.08513059	2.968651	0.2193572	0.7644557
Responder	ECT	0.3984938	0.11525760	7.907904	0.1321696	0.6648181

13.7.1. Pairwise Contrasts (Tukey): Response by ECT

contrast	ECT	estimate	SE	df	t_ratio	p_value
NO - YES	No ECT	-0.003439895	0.08260719	123.95342	-0.04164159	0.9668514
NO - YES	ECT	0.093412629	0.09950390	99.29346	0.93878360	0.3501199

13.8. Estimated Marginal Means (EMMs): ECT by Response

ECT	Response	EMM	SE	df	CI_low	CI_high
No ECT	Non-responder	0.5400835	0.07624500	1.954143	0.2045390	0.8756280
ECT	Non-responder	0.4919065	0.08513059	2.968651	0.2193572	0.7644557
No ECT	Responder	0.5435234	0.10835200	6.188397	0.2803399	0.8067069
ECT	Responder	0.3984938	0.11525760	7.907904	0.1321696	0.6648181

13.8.1. Pairwise Contrasts (Tukey): ECT by Response

contrast	Response	estimate	SE	df	t_ratio	p_value
NO - YES	Non-responder	0.04817702	0.04634770	102.2638	1.039470	0.30103779
NO - YES	Responder	0.14502955	0.07876521	116.2431	1.841289	0.06812882

14. Supplementary Results: Comorbidities				
**Comorbidity category**	**UDC (n=36)**	**TRD-M (n=82)**	**TRD-ECT (n=78)**	**p-value**
**Somatic comorbidities**				
Diseases of the circulatory system	11.1%	23.1%	39.7%	0.003
Endocrine, nutritional and metabolic diseases	2.8%	24.4%	28.2%	0.130
Diseases of the respiratory system	2.8%	13.4%	9.0%	0.191
Diseases of the nervous system	0.0%	15.9%	14.1%	0.132
**Psychiatric comorbidities (secondary diagnoses)**				
F00–F09: Organic, including symptomatic, mental disorders	0.0%	1.2%	1.3%	0.796
F10–F19: Mental disorders due to psychoactive substance use	8.3%	22.0%	21.8%	0.178
F20–F29: Schizophrenia and related disorders	0.0%	1.2%	2.6%	0.559
F30–F39: Mood disorders	0.0%	6.1%	3.8%	0.302
F40–F49: Neurotic, stress-related and somatoform disorders	41.7%	42.7%	34.6%	0.550
F50–F59: Behavioral syndromes associated with physiological disturbances	8.3%	8.5%	5.1%	0.672
F60–F69: Disorders of adult personality and behavior	11.1%	28.0%	15.4%	0.046
F70–F79: Intellectual disabilities	0.0%	0.0%	0.0%	-
F80–F89: Disorders of psychological development	2.8%	1.2%	2.6%	0.786
F90–F99: Behavioral and emotional disorders with onset in childhood/adolescence	2.8%	7.3%	5.1%	0.597
